# SARS-CoV-2 mRNA vaccination–induced immunological memory in human nonlymphoid and lymphoid tissues

**DOI:** 10.1172/JCI171797

**Published:** 2023-12-15

**Authors:** Vanessa Proß, Arne Sattler, Sören Lukassen, Laura Tóth, Linda Marie Laura Thole, Janine Siegle, Carolin Stahl, An He, Georg Damm, Daniel Seehofer, Christina Götz, Christian Bayerl, Pia Jäger, Alexander Macke, Stephan Eggeling, Bernadette Kirzinger, Thomas Mayr, Hermann Herbst, Katharina Beyer, Dominik Laue, Jan Krönke, Jan Braune, Friederike Rosseck, Beatrice Kittner, Frank Friedersdorff, Mandy Hubatsch, Sarah Weinberger, Nils Lachmann, Veit Maria Hofmann, Eva Schrezenmeier, Carolin Ludwig, Hubert Schrezenmeier, Katharina Jechow, Christian Conrad, Katja Kotsch

**Affiliations:** 1 Department of General and Visceral Surgery, Charité – Universitätsmedizin Berlin, Corporate Member of Freie Universität Berlin, Humboldt-Universität zu Berlin, and Berlin Institute of Health, Berlin, Germany.; 2 Center of Digital Health, Berlin Institute of Health and Charité – Universitätsmedizin Berlin, Corporate Member of Freie Universität Berlin and Humboldt-Universität zu Berlin, Berlin, Germany.; 3 Department of Hepatobiliary Surgery and Visceral Transplantation, University Hospital, Leipzig University, Leipzig, Germany.; 4 Department of Radiology, Charité – Universitätsmedizin Berlin, Campus Benjamin Franklin, Corporate Member of Freie Universität Berlin, Humboldt-Universität zu Berlin, and Berlin Institute of Health, Berlin, Germany.; 5 Department of Visceral Surgery,; 6 Department of Thoracic Surgery, and; 7 Department of Pathology, Vivantes Klinikum Neukölln, Berlin, Germany.; 8 Department of Traumatology and Reconstructive Surgery, Campus Benjamin Franklin, Charité – Universitätsmedizin Berlin, Corporate Member of Freie Universität Berlin, Humboldt-Universität zu Berlin, and Berlin Institute of Health, Berlin, Germany.; 9 Department of Hematology, Oncology and Cancer Immunology, Charité – Universitätsmedizin Berlin, Corporate Member of Freie Universität Berlin and Humboldt-Universität zu Berlin, Berlin, Germany.; 10 Institute of Pathology, Charité – Universitätsmedizin Berlin, Corporate Member of Freie Universität Berlin, Humboldt-Universität zu Berlin, and Berlin Institute of Health, Berlin, Germany.; 11 Department of Urology, Evangelisches Krankenhaus Königin Elisabeth Herzberge, Berlin, Germany.; 12 Department of Urology, Charité – Universitätsmedizin Berlin, Corporate Member of Freie Universität Berlin, Humboldt-Universität zu Berlin, and Berlin Institute of Health, Berlin, Germany.; 13 Institute of Transfusion Medicine, Berlin Institute of Health, Charité – Universitätsmedizin Berlin, Humboldt-Universität zu Berlin, Berlin, Germany.; 14 Department of Otolaryngology, Charité – Universitätsmedizin Berlin, Corporate Member of Freie Universität Berlin, Humboldt-Universität zu Berlin, and Berlin Institute of Health, Berlin, Germany.; 15 Department of Nephrology and Medical Intensive Care, Charité – Universitätsmedizin Berlin, Corporate Member of Freie Universität Berlin and Humboldt-Universität zu Berlin, Berlin, Germany.; 16 BIH Charité Clinician Scientist Program, BIH Biomedical Innovation Academy, Berlin Institute of Health at Charité – Universitätsmedizin Berlin, Berlin, Germany.; 17 Institute for Clinical Transfusion Medicine and Immunogenetics, German Red Cross Blood Transfusion Service Baden-Württemberg-Hessen and University Hospital Ulm, Ulm, Germany.

**Keywords:** Immunology, Vaccines, Adaptive immunity, T cells

## Abstract

Tissue-resident lymphocytes provide organ-adapted protection against invading pathogens. Whereas their biology has been examined in great detail in various infection models, their generation and functionality in response to vaccination have not been comprehensively analyzed in humans. We therefore studied SARS-CoV-2 mRNA vaccine–specific T cells in surgery specimens of kidney, liver, lung, bone marrow, and spleen compared with paired blood samples from largely virus-naive individuals. As opposed to lymphoid tissues, nonlymphoid organs harbored significantly elevated frequencies of spike-specific CD4^+^ T cells compared with blood showing hallmarks of tissue residency and an expanded memory pool. Organ-derived CD4^+^ T cells further exhibited increased polyfunctionality over those detected in blood. Single-cell RNA-Seq together with T cell receptor repertoire analysis indicated that the clonotype rather than organ origin is a major determinant of transcriptomic state in vaccine-specific CD4^+^ T cells. In summary, our data demonstrate that SARS-CoV-2 vaccination entails acquisition of tissue memory and residency features in organs distant from the inoculation site, thereby contributing to our understanding of how local tissue protection might be accomplished.

## Graphical abstract

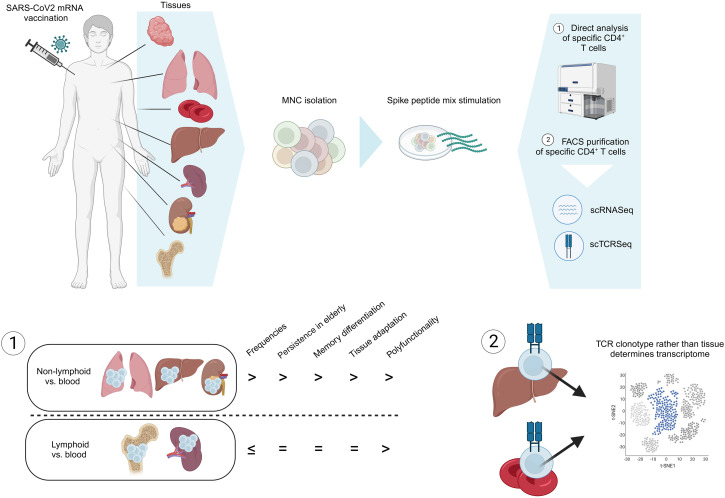

## Introduction

Vaccination against SARS-CoV-2 has been proven in large cohort studies (1, 2) to be a powerful strategy to protect from severe coronavirus disease 2019 (COVID-19). Since the availability of the first SARS-CoV-2 vaccines, specific humoral and cellular immunity has been characterized in unprecedented depth in healthy individuals and those with multiple preexisting conditions (3–5). Whereas infection- or vaccination-induced T cell biology has been comprehensively examined in peripheral blood, only limited information is available as to how immunological memory is established in tissues. Postulated more than 10 years ago, the concept of tissue residency has been raised in the contexts of different tissue-targeted infections, implying that a substantial proportion of memory T cells have a limited capacity to recirculate but acquire residency in organs, thus providing site-adapted immunity (6–8). Pioneering work noted a distribution of SARS-CoV-2 infection–induced T cells across multiple human tissues, including lymph nodes, spleen, lung, and bone marrow. A dichotomy for tissue residency–related molecules was identified between virus-specific CD8^+^ and CD4^+^ T cells, with the latter showing coexpression of the signature markers CD69 and CD103 almost exclusively in the lung (9). Multi-organ residency is also characteristic of cytomegalovirus-specific T cells (10), which have recently been detected in high frequencies, along with those specific for Epstein-Barr virus and influenza, in resected human kidneys (11).

With respect to features of vaccination-induced T cell memory, most comprehensive analyses in organs have been conducted using human bone marrow, where CD4^+^ T cells specific for multiple vaccination-associated antigens, including tetanus toxoid, measles, and mumps, have been characterized (12). In comparison with peripheral blood, CD4^+^ T helper (Th) cells were in a resting state and upregulated CD69, both being indicative of bone marrow as a niche for long-term memory for systemic pathogens (13). It remains to be determined how SARS-CoV-2 mRNA vaccination–induced T cell memory is maintained with respect to organ tropism and tissue adaptation, particularly considering nonlymphoid organs. Both features might be potentially influenced by in vivo distribution of vaccination antigens. For novel mRNA-based vaccines, distribution and degradation of encoded proteins have been followed in experimental models. After intramuscular application, protein expression was predominantly confined to the injection site, but also included distal organs such as lung and liver, indicating systemic spread of mRNA-containing lipid nanoparticles (14). Similar findings regarding biodistribution were made for a vector-based SARS-CoV-2 vaccine (AZD1222/ChAdOx1), detectable for up to 29 days in bone marrow, liver, lung, spleen, and lymph nodes (15). It is therefore conceivable that differentiation and/or recruitment of cellular immunity involves organs distant to the vaccination site. To address tissue distribution of SARS-CoV-2 vaccine–specific T cell memory, we examined human lymphoid (bone marrow, spleen, tonsil) and nonlymphoid (liver, kidney, lung) organs for quantities and functional features of spike protein–specific T cells in comparison with paired blood samples. Here, we identify mRNA vaccine–specific CD4^+^ T cells in most tissue types examined with distinct adaptations particularly identified for nonlymphoid organs.

## Results

### Donor tissue cohort.

**Figure 1:**
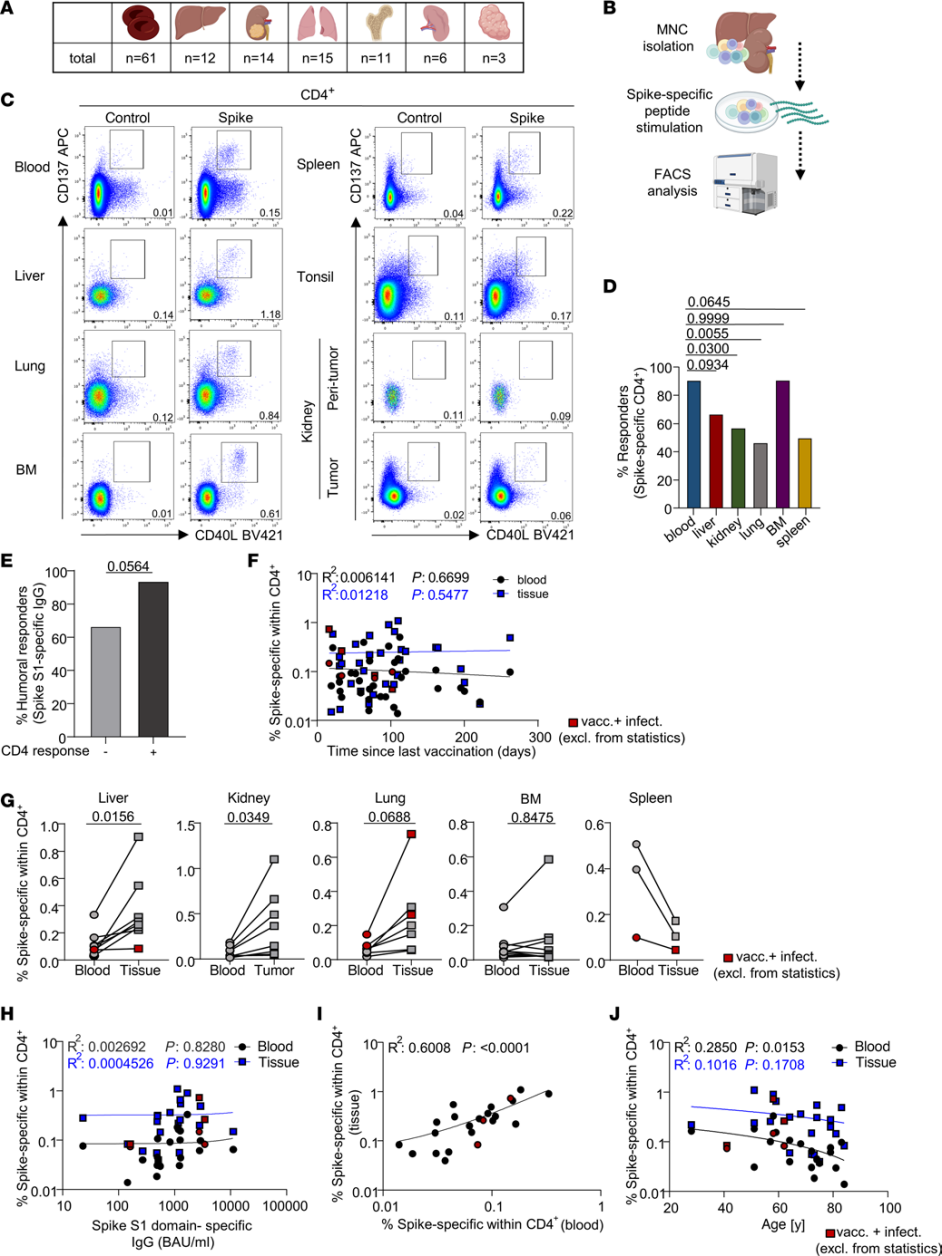
Quantification of SARS-CoV-2 vaccine–induced CD4^+^ Th cells in nonlymphoid and lymphoid organs. (**A**) Summary of all specimens included for analysis of vaccine-specific T cells. (**B**) Schematic workflow overview. (**C**) Exemplary plots showing vaccine-specific CD137^+^CD40L^+^ CD4^+^ T cells from the indicated organs as identified by FACS. (**D**) Proportions of individuals showing spike-specific CD4^+^ T cell responses within the depicted organs. Statistically significant differences were tested with 2-sided Fisher’s exact test with *n* as in **A**. (**E**) Proportions of individuals with spike S1 domain–specific IgG responses, stratified for cellular responders and nonresponders. Statistically significant differences were tested with 2-sided Fisher’s exact test. (**F**) Simple linear regression analysis between frequencies of spike-specific Th cells and time since last vaccination with *n* as in **A**. (**G**) Pairwise comparison of spike-specific CD4^+^ T cell frequencies in peripheral blood–derived and organ-derived specimens as indicated. Liver: *n* = 8, Wilcoxon’s test; kidney: *n* = 8, paired *t* test; lung: *n* = 7, paired *t* test; bone marrow: *n* = 10, Wilcoxon’s test; spleen: *n* = 3, paired *t* test. (**H** and **I**) Simple linear regression analysis between frequencies of specific Th cells in nonlymphoid organs and spike S1 domain–specific IgG levels (**H**) or paired blood samples (**I**). BAU, binding antibody units. (**J**) Simple linear regression analysis between specific blood-derived and paired nonlymphoid organ–derived T cell frequencies and age. Red symbols identify vaccinated individuals with a history of SARS-CoV-2 infection that were excluded from statistics.

To analyze SARS-CoV-2 vaccine–specific T and B cells in various human tissues, specimens of nonlymphoid (liver, kidney, lung) as well as lymphoid (bone marrow, spleen, tonsil) organs were procured together with paired blood (Figure 1A). Surgeries were primarily, but not exclusively, conducted for tumor resection; in these cases, peritumor tissue located most distant to the tumor was used unless otherwise indicated. To focus on vaccination-induced immunity, the majority of individuals enrolled were SARS-CoV-2 naive as evidenced by medical history and absence of reactivity in a SARS-CoV-2 nucleocapsid–specific ELISA. Details on patient demographics are summarized in Table 1. Tissue, blood, and serum samples were immediately processed after collection and cryopreserved before assessment of vaccine-specific immunity (Figure 1B). Depending on the time point of sample procurement, vaccination history of individuals comprised 2 or 3 mRNA vaccine doses (BioNTech/Pfizer or Moderna) (Table 1).

### SARS-CoV-2 vaccine–specific T cells in nonlymphoid and lymphoid tissues.

Specific CD4^+^ Th cells were identified based on CD137 and CD40L coexpression after stimulation with an overlapping peptide pool encompassing the complete SARS-CoV-2 spike protein as outlined in Supplemental Figure 1A (supplemental material available online with this article; https://doi.org/10.1172/JCI171797DS1). A response was defined as positive when stimulated mononuclear cells (MNCs) contained at least 2-fold higher frequencies of CD137^+^CD154^+^ CD4^+^ T cells (also applying to CD137^+^IFN-γ^+^ CD8^+^ T cells) as compared with the respective unstimulated control with at least 20 events, as reported previously (4, 16). To assess ex vivo expressing, but not stimulation-induced, CD69^+^ T cells, MNCs were stained with CD69-BV785 before culture; labeling was stable without appreciable loss of signal intensity until stimulation termination as recently demonstrated (11) and as depicted in Supplemental Figure 1B. Overall, spike-specific CD4^+^ T cells could be identified in peripheral blood, liver, lung, bone marrow, spleen, and kidney tumor but not in kidney peritumor tissue or tonsil as exemplified in Figure 1C. Among all samples, cellular responses were most frequently detected in blood and bone marrow (Figure 1D). Individuals with cellular responses in peripheral blood showed a trend toward an elevated rate of specific IgG responses over cellular nonresponders (Figure 1E). In most analyses, for rough comparison with vaccinated-only probands, data of some infected plus vaccinated individuals were included. They were, however, excluded from statistics.

To verify that our virus-naive cohort did not contain individuals with an unreported SARS-CoV-2 infection, we conducted peptide mix stimulations representing SARS-CoV-2 membrane and nucleocapsid protein (termed “M+N”) at random with remaining samples. Supplemental Figure 2A (left) shows exemplary CD4 responses in PBMCs from individuals considered virus-naive + vaccinated versus infected + vaccinated after M+N and spike stimulation, with a summary depicted in Supplemental Figure 2A (right). Results highlight that all 4 of 4 individuals with documented infection could be identified based on the M+N assay. Importantly, none of the supposedly noninfected patients showed M+N reactivity. These random controls support our data from medical records and serology indicating that we did not accidentally include individuals with a SARS-CoV-2 infection history in this group.

Frequencies of spike-specific Th cells (Figure 1F) and those exhibiting a memory or effector phenotype or expressing IFN-γ or IL-2 (Supplemental Figure 2B) remained constant in blood and tissue with progressing time from last vaccination. Similarly, no significant differences were identified with respect to cellular responder rates (Supplemental Figure 3A), frequencies, memory differentiation, or function (Supplemental Figure 3B) between tumor and nontumor patient-derived blood samples, excluding an appreciable impact of patients’ preexisting conditions.

For subsequent analyses, comparisons between paired specimens were conducted when both blood and tissue samples fulfilled the criteria for a cellular response. As a common motif, nonlymphoid (liver, kidney tumor, lung) but not lymphoid (bone marrow, spleen) tissues were characterized by significantly elevated frequencies (or a clear trend thereof in the case of lung) of spike-specific CD4^+^ T cells over those quantified in blood (Figure 1G). We did not note a significant correlation between spike-specific IgG levels and frequencies of blood- or nonlymphoid tissue–derived CD4^+^ T cells (Figure 1H). However, frequencies of specific CD4^+^ T cells detected in blood significantly correlated with those in paired nonlymphoid organs (Figure 1I) and showed a trend for lymphoid organs (Supplemental Figure 4A). Interestingly, proportions of spike-specific CD4^+^ T cells significantly declined with age in blood, but not in paired nonlymphoid tissues (Figure 1J). The observation did not apply to paired blood of lymphoid tissues (Supplemental Figure 4B), obviously owing to the smaller sample size. In summary, vaccine-specific CD4^+^ Th cell responses could be detected in both lymphoid and nonlymphoid organs. Only for the latter, we observed an enrichment of specific cells in tissue-derived compared with blood-derived specimens.

### Memory differentiation and tissue adaptation of vaccine-specific CD4^+^ T cells.

**Figure 2:**
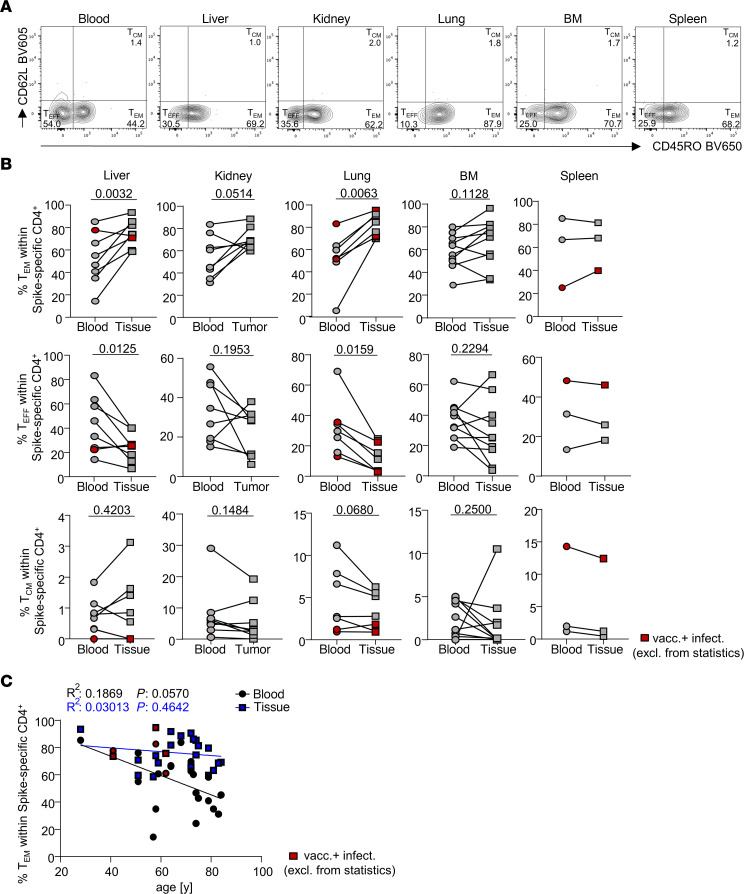
Enrichment of specific memory-type CD4^+^ T cells in nonlymphoid tissues. (**A** and **B**) Exemplary plots (**A**) for FACS-based identification of CD45RO^+^CD62L^–^ memory (Tm) and CD45RO^–^CD62L^–^ effector-type (Teff) T cells within the spike-specific compartment of different paired samples as summarized in **B**. Liver: *n* = 8, paired *t* test; kidney: *n* = 8, paired *t* test for Tm and Wilcoxon’s test for Teff; lung: *n* = 7, paired *t* test; bone marrow: *n* = 10, paired *t* test; spleen: *n* = 3, paired *t* test. (**C**) Simple linear regression analysis between specific blood-derived and paired nonlymphoid organ–derived Tm cell frequencies and age. Red symbols identify vaccinated individuals with a history of SARS-CoV-2 infection that were excluded from statistics.

To identify distinct organ-specific adaptation patterns, vaccine-specific CD4^+^ lymphocytes were further characterized according to expression of typical molecules reflecting memory phenotype (CD45RO, CD62L) and tissue residency (CD69, CD103, CD49a). Nonlymphoid organs were enriched for specific CD45RO^+^CD62L^–^ effector-memory-type T (Tem) cells, along with a drop in CD45RO^–^CD62L^–^ effector-type (Teff) cells, as compared with paired blood; the Tem pattern also showed a similar trend for bone marrow, but not for spleen, also owing to sample size. A minority of specific T cells in all tissues belonged to the CD45RO^+^CD62L^+^ central memory (Tcm) subset (Figure 2, A and B). Frequencies of antigen-specific Tem cells declined according to age in peripheral blood, but not in nonlymphoid tissue (Figure 2C). This dichotomy was not evident for lymphoid tissue and paired blood specimens, again likely owing to the smaller sample size (Supplemental Figure 4C). The predominance of specific effector-memory-type Th cells in most tissues based on CD45RO/CD62L expression was corroborated by staining of the alternative marker combination CD45RA/CCR7, similarly identifying most cells, with few exceptions, as CD45RA^–^CCR7^–^ effector memory cells. For further comparison, the combination of CD45RO/CCR7 was exemplarily included for one set of specimens (Supplemental Figure 4D).

**Figure 3:**
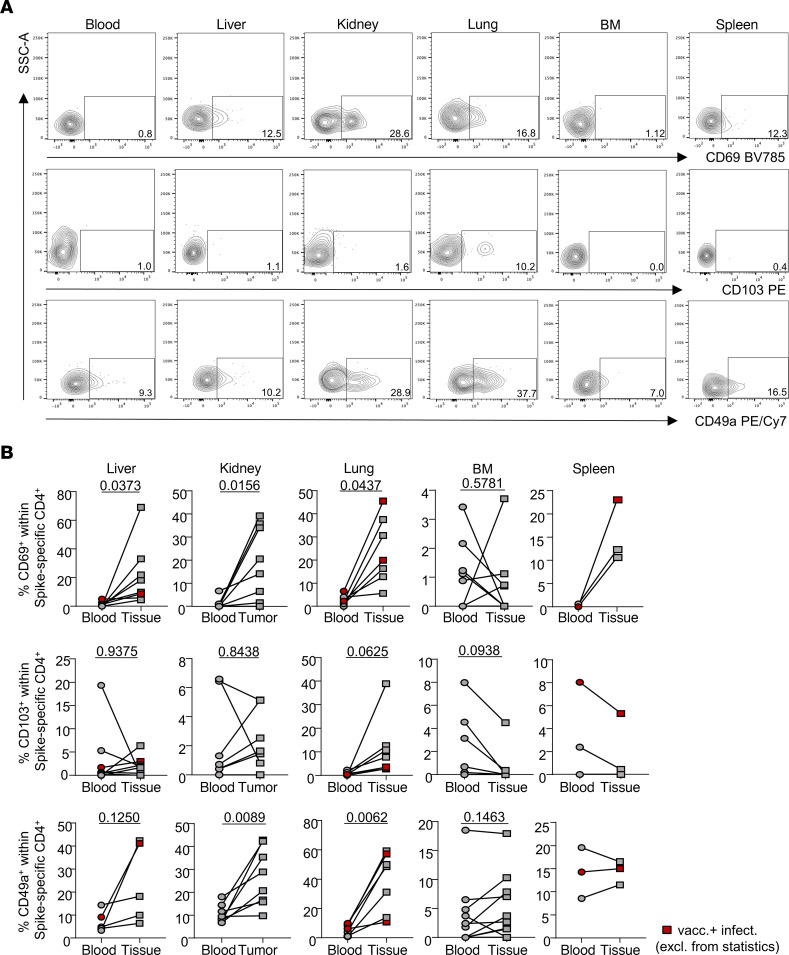
Tissue adaptation signatures of vaccine-specific CD4^+^ T cells. (**A** and **B**) Exemplary plots (**A**) and summary (**B**) for FACS-based identification of the tissue residency/retention–associated molecules CD69, CD103, and CD49a among vaccine-specific CD4^+^ T cells in the indicated specimen types. Liver: *n* = 5, paired *t* test for CD69 and Wilcoxon’s test for CD103/CD49a; kidney: *n* = 8, Wilcoxon’s test for CD69/CD103 and paired *t* test for CD49a; lung: *n* = 7, paired *t* test for CD69/CD49a and Wilcoxon’s test for CD103; bone marrow: *n* = 10, Wilcoxon’s test for CD69/CD103 and paired *t* test for CD49a; spleen: *n* = 3, Wilcoxon’s test for CD69 and paired *t* test for CD103/CD49a. Red symbols identify vaccinated individuals with a history of SARS-CoV-2 infection that were excluded from statistics.

Notably, a similar segregation between both organ systems could be observed based on frequencies of CD69^+^ and CD49a^+^ cells that tended to be, or were significantly, elevated in nonlymphoid but not in lymphoid organs compared with paired blood samples with the exception of CD69 in spleen (Figure 3, A and B). Interestingly, the integrin CD103 was only detectable in a minor proportion of antigen-experienced CD4^+^ T cells, and expression was mainly confined to the lung.

### Polyfunctionality as a distinct feature of spike-specific Th cells from organs.

**Figure 4:**
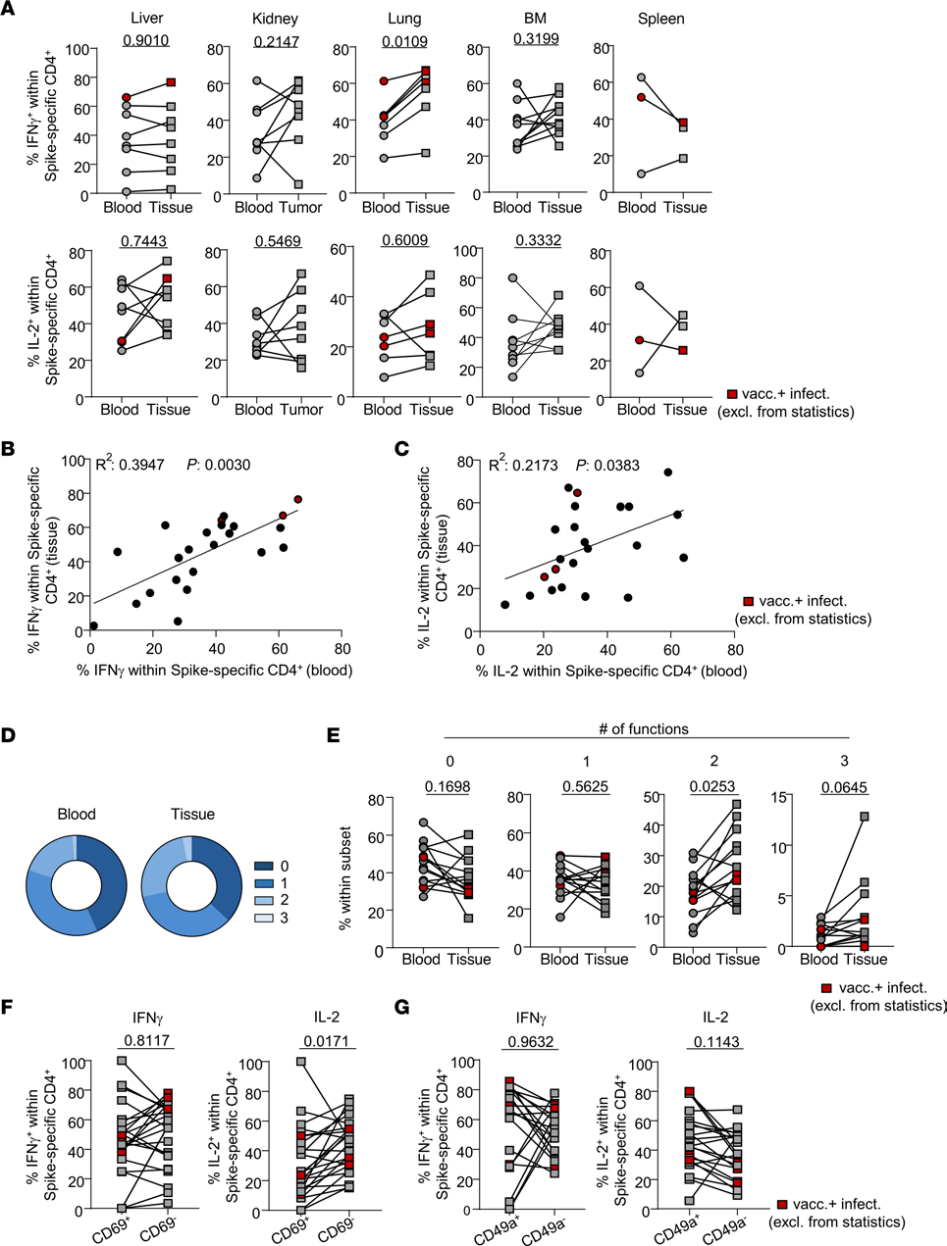
Enhanced polyfunctionality as a feature of specific organ-derived Th cells. Cytokine expression was assessed in spike-specific Th cells intracellularly by FACS. (**A**) Frequencies of IFN-γ– or IL-2–positive cells among the indicated paired samples. Liver: *n* = 8, paired *t* test; kidney: *n* = 8, paired *t* test for IFN-γ and Wilcoxon’s test for IL-2; lung: *n* = 7, paired *t* test; bone marrow: *n* = 10, paired *t* test; spleen: *n* = 3, paired *t* test. (**B** and **C**) Simple linear regression analysis of frequencies of specific IFN-γ–expressing (**B**) or IL-2–expressing (**C**) Th cells from nonlymphoid tissues versus paired blood. (**D** and **E**) Mean frequencies (**D**) and paired analyses (**E**) of spike-specific polyfunctional Th cells expressing 3, 2, 1, or 0 of the cytokines IFN-γ, IL-2, and/or IL-4 at a time. Statistically significant differences were tested with paired *t* test (0–2 cytokines) or with Wilcoxon’s test (3 cytokines). (**F** and **G**) Differential IFN-γ or IL-2 expression in spike-specific Th cells from nonlymphoid organs after pre-gating on CD69^+^ or CD69^–^ (**F**) and CD49a^+^ or CD49a^–^ (**G**) expressing or nonexpressing subsets. Liver: *n* = 8; kidney: *n* = 8; lung: *n* = 7. Statistically significant differences were tested with paired *t* test (IL-2) or with Wilcoxon’s test (IFN-γ). For **D**–**G**, only tissue samples from nonlymphoid organs were included. Red symbols identify vaccinated individuals with a history of SARS-CoV-2 infection that were excluded from statistics.

To test the hypothesis that tissue-derived, vaccine-specific CD4^+^ T cells show enhanced functionality as compared with those detected in blood, cytokine production was assessed by FACS (Supplemental Figure 5). No significant differences were detected in frequencies of IL-2–, IL-4–, and IFN-γ–producing cells except in lung, which showed elevated frequencies of IFN-γ^+^ T cells in tissue over blood (Figure 4A and Supplemental Figure 6A). Frequencies of IL-2– and IFN-γ–positive cells correlated between nonlymphoid tissues and paired blood samples (Figure 4, B and C), which could not be verified for lymphoid tissues (Supplemental Figure 6, B and C). Interestingly, we determined polyfunctionality as a key characteristic separating blood- from organ-derived T cells. Nonlymphoid organs were enriched for cells expressing 2 or 3 cytokines at a time (Figure 4, D and E), with only enrichment of triple producers being equally observed for lymphoid organ–derived cells (Supplemental Figure 6, D and E).

Further investigation revealed an enrichment of specific IL-2–producing, but not IFN-γ–producing, Th cells within the CD69^–^ subpopulation. On the contrary, IL-2 producers showed a trend toward enrichment in the CD49a^+^ Th subset (Figure 4, F and G, and Supplemental Figure 6F). These analyses were solely conducted for nonlymphoid organs given the paucity of CD69 and/or CD49a expression in lymphoid organs as demonstrated in Figure 3, A and B.

Overall, specific Th cells in both blood and all tissues showed a skewing toward Th1 as reflected by dominant coexpression of IFN-γ/IL-2 over IL-4/IL-2 (Supplemental Figure 7, A and B). Assessment of the global capacity of spike-specific CD4^+^ T cells to produce at least 1 of the 3 cytokines IFN-γ, IL-2, and IL-4 revealed no significant differences between tissues and paired blood samples (Supplemental Figure 7C).

In summary, organ-derived T cells show functional superiority to their blood-derived counterparts mirrored by increased quantities of multipotent cells.

### Vaccine-specific CD8^+^ T cells in nonlymphoid and lymphoid tissues.

Along with their CD4^+^ counterparts, antigen-specific CD8^+^ T cells were identified within the same samples according to CD137 and IFN-γ coexpression, as recently shown (4, 16) with the gating strategy depicted in Supplemental Figure 1. Our approach to use 15-mers with 11–amino acid overlap for stimulation of both CD4^+^ and CD8^+^ T cells might slightly less efficiently activate the latter, but has been determined a good compromise when measuring both responses within 1 sample (17). CD8 responder rates, particularly in peripheral blood, were consistently lower as compared with their CD4^+^ counterparts (Supplemental Figure 8A). We observed a pattern similar to that seen for CD4 responses in that spike-specific CD8^+^ T cells were detectable in most organ types with the exception of peritumor kidney tissue and tonsil, where response criteria were consistently not met (Supplemental Figure 8B). Within a limited set of paired samples, no significant elevation of frequencies was observed in tissues over blood (Supplemental Figure 8C). With respect to CD45RO and CD62L expression, no clear sample-type-specific pattern was evident for spike-specific CD8^+^ T cells with the exception of liver tissue showing an enrichment of memory-type T cells over blood (Supplemental Figure 9). Furthermore, although statistical analyses were not adequate due to limited sample size, nonlymphoid organs tended to show selective enrichment of specific CD69^+^, CD103^+^, and/or CD49a^+^ CD8^+^ T cells compared with blood (Supplemental Figure 9).

### SARS-CoV-2 vaccination–induced memory B cells are detectable in tissues.

Within a limited set of peripheral blood and tissue specimens we sought to characterize quantities and phenotype of vaccine-specific B cells with the gating strategy depicted in Supplemental Figure 10. Importantly, spike-specific B cells were detectable in all organ and blood samples (specimen types summarized in Supplemental Figure 11A and exemplary stainings in Supplemental Figure 11B, top) with frequencies typically ranging between 0.1% and 0.01% within the CD19^+^ compartment (Supplemental Figure 11, B, top, and C, left). Overall, portions of isotype class–switched IgD^–^CD27^+^ memory cells constituted the majority of specific B cells within lymphoid organs and blood, but not in kidney (Supplemental Figure 11, B, middle, and C, middle). Expression of CD69 as a marker for tissue retention (18) was confined to minor proportions of spike-specific B cells of most specimens with few individual exceptions (Supplemental Figure 11, B, bottom, and C, right). Subanalysis of specific IgD^–^CD27^+^ memory B cells revealed, depending on specimen type, varying proportions of IgG^+^ cells (Supplemental Figure 11D). Given the small sample size and, in some subanalyses, less than 10 specific cells, statistical analyses were not conducted.

### Single-cell transcriptomics of vaccine-specific CD4^+^ Th cells.

**Figure 5:**
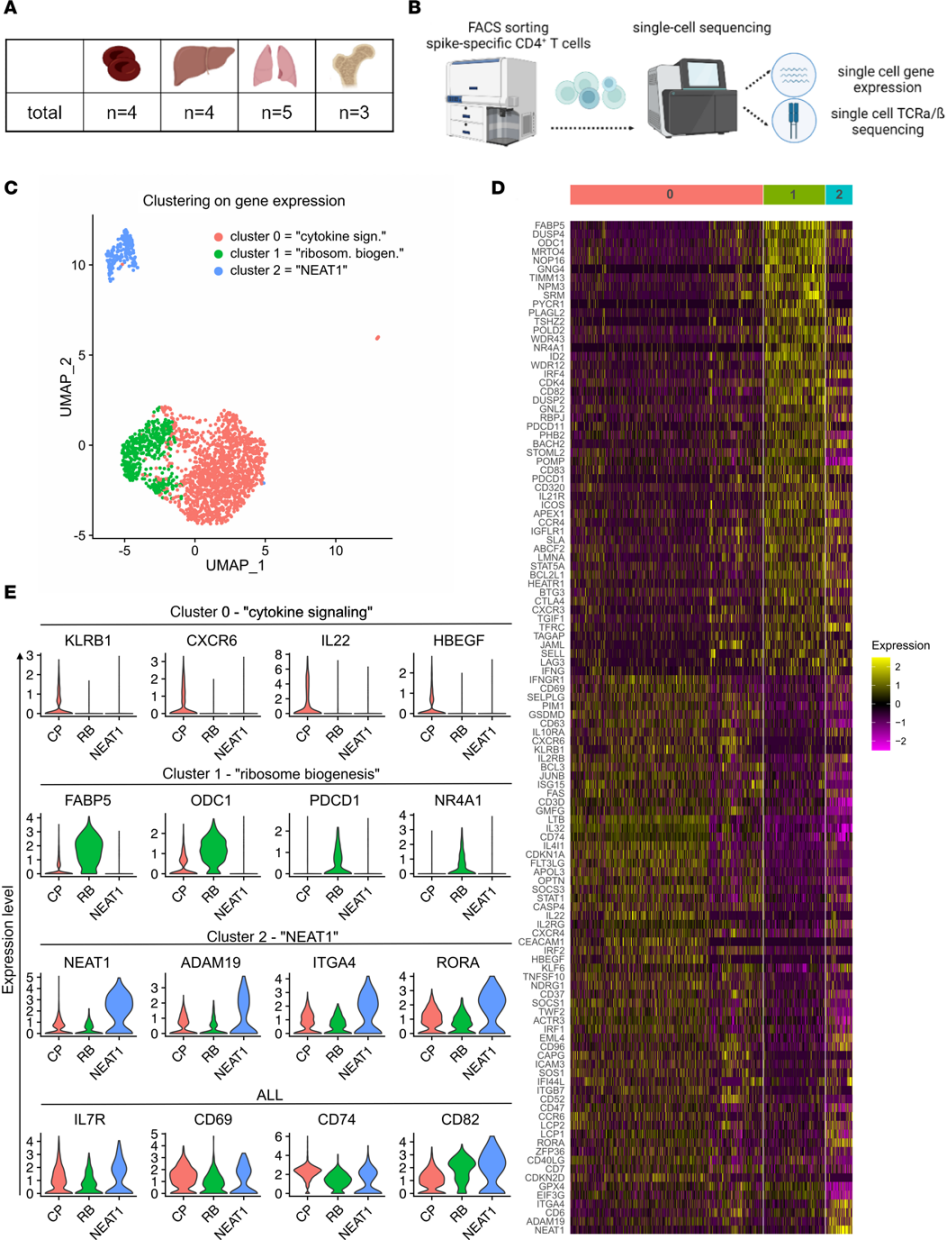
scRNA-Seq analysis of spike-specific Th cells from organs and blood. (**A** and **B**) Summary of specimens included (**A**) and workflow for transcriptome analysis of spike-specific CD4^+^ Th cells (**B**). (**C**) Unsupervised clustering based on transcriptomes derived from *n* = 1,985 cells identified 3 major populations when visualized by UMAP. (**D**) Heatmap showing expression patterns of selected characteristic genes for clusters 0, 1, and 2. (**E**) Violin plots displaying selection of genes that are differentially regulated in clusters 0 (first panel), 1 (second panel), and 2 (third panel) and those that are similarly regulated over all clusters (fourth panel).

Activation marker–based isolation of SARS-CoV-2–specific T cells after antigen-specific stimulation, followed by single-cell RNA sequencing (scRNA-Seq), has already been employed to obtain a deeper understanding of virus-specific, tissue-resident CD8^+^ T cells (19). Using this approach, vaccine-specific CD137^+^CD40L^+^ cells from peripheral blood (*n* = 4), liver (*n* = 4), lung (*n* = 5), and bone marrow (*n* = 3) were FACS-sorted to purities greater than 97 % after peptide stimulation (Figure 5A and Supplemental Figure 1), followed by transcriptome assessment (Figure 5B). Blood and liver samples were derived from the same 4 patients, whereas all other specimens were from different individuals. After quality filtering (Supplemental Figure 12), unsupervised clustering of 1,985 spike-specific Th cells yielded 3 clusters visualized as uniform manifold approximation and projection (UMAP) (Figure 5C) for which pathway enrichment analyses were conducted (Supplemental Figure 13). Cluster 0 was characterized by the upregulation of cytokine signaling–related pathways (termed “cytokine signaling”), whereas cluster 1 showed an enrichment of ribosomal biogenesis–related genes (“ribosomal biogenesis”). Cluster 2, showing the most pronounced separation in UMAP, was enriched for developmental, cell adhesion–related, and T cell activation pathways with the long noncoding RNA *NEAT1* as the most upregulated gene within this cluster (“NEAT1”).

**Figure 6:**
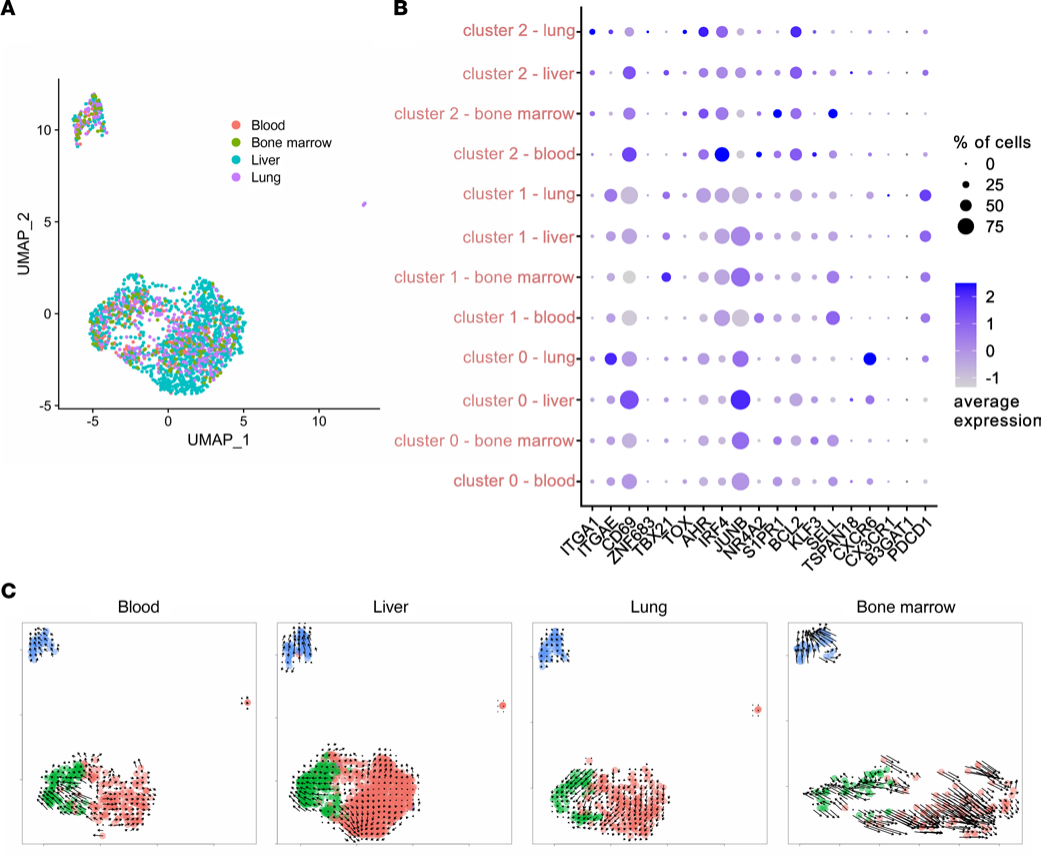
Cell clustering is not primarily driven by tissue-specific features. (**A**) UMAP plot as in Figure 5C with overlay of specimen origins. (**B**) Expression of selected tissue residency/retention–associated or –nonassociated genes in cells derived from distinct cluster/tissue combinations. Expression values are shown as *z* scores. (**C**) Grid representation of RNA velocities for the various tissues calculated using velocyto. Data sets were split according to tissue before velocity calculation, and cells are color-coded by cluster.

Genes related to tissue homing and residency, including *KLRB1* and the chemokine receptor *CXCR6* (20–22), were solely upregulated in cluster 0. This cluster showed further transcript enrichments for proinflammatory mediators including IL-22, as well as for heparin-binding EGF (HBEGF), a marker suggested to shape antigen-specific CD4^+^ T cell responses and constrain Th17 differentiation (23). Upregulated genes involved in various metabolic pathways were observed in cluster 1, including *FABP5* and *ODC1*, both involved in lipid metabolism of tissue-resident lymphocytes (24, 25). This cluster was also characterized by induction of the tissue residency transcript *PDCD1*, which has been suggested as a feature of murine tissue-resident brain cells (26, 27), and the transcription factor *NRF4A1*, important for controlling tissue retention (28). In contrast, genes encoding products involved in tissue-resident cell activation, migration, or retention, including *ADAM19* (29), the integrin *ITGA4*, and the T cell lineage regulator *RORA* (30), were identified for cluster 2. Several transcripts involved in memory differentiation/tissue retention or activation were similarly expressed across clusters, including *IL7R*, *CD69*, *CD74*, and *CD82* (Figure 5, D and E). Surprisingly, using UMAP, cells from different tissues were not selectively associated with but were evenly distributed over all 3 clusters (Figure 6A). This observation is in line with the fact that transcripts for a selected set of typical tissue-related (e.g., *ITGAE*, *ZNF683*, *CXCR6*) or circulation/migration-related genes (e.g., *S1PR1*, *SELL*) were cluster- rather than organ-specific (Figure 6B). RNA velocity analysis for the various tissues revealed some transcriptional dynamics within, but not across, clusters, indicating that the cluster identity itself was likely static, an observation that generally holds for the individual organs (Figure 6C).

### T cell receptor analysis of SARS-CoV-2 vaccine–specific T cells.

**Figure 7:**
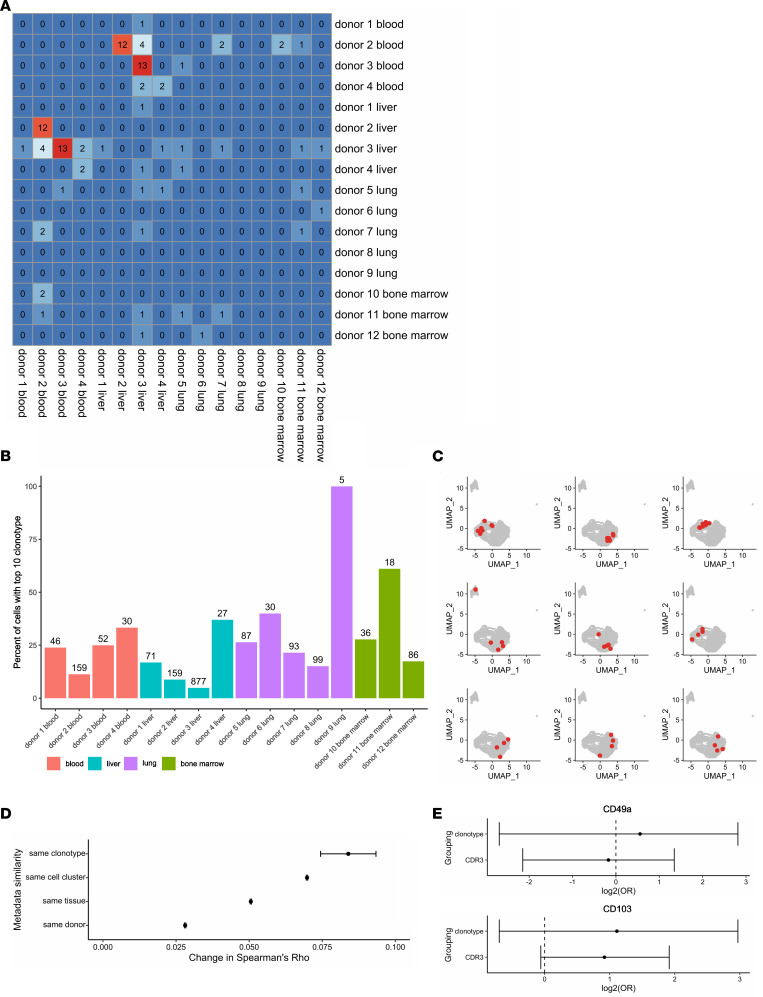
Shared TCR clonotypes between tissues. (**A**) Heatmap depicting the overlap in absolute numbers of CDR3 sequences in different samples. (**B**) Percentage of cells with at least 1 of the 10 most frequent clonotypes per sample, colored by organ. Total cell numbers with known clonotype are indicated above the bars. Blood and liver samples from donors 1–4 were paired, whereas samples 5–12 were from different donors. (**C**) Association of clonotypes with gene expression. UMAP plots with cells that have a shared clonotype highlighted in red. Separate graphs for all 9 different clonotypes with at least 4 cells (inclusion criterion) are shown. (**D**) Impact of shared versus different metadata on the cell-cell Spearman’s correlation coefficient for highly variable genes. Mean change and 95% confidence intervals were obtained using Tukey’s honestly significant difference test, considering all individual variables as well as their interactions. (**E**) log_2_ odds ratio for clonotypes or CDR3 sequences shared between blood and liver in liver-derived cells positive versus negative for CD49a (top) or CD103 (bottom). Positivity for these markers was defined as the presence of at least 1 count of the respective molecule. Whiskers extend to the 95% confidence interval.

For 1,875 of 1,985 sequenced cells, a T cell receptor (TCR) clonotype could be obtained. As the sequenced T cells should have a similar antigen specificity, we were interested in the degree of overlap of complementarity-determining region 3 (CDR3) sequences between different individuals, as well as different tissues from the same individual. As expected, the highest number of overlapping CDR3 sequences was observed between blood and liver within the same individual (blood/liver donors 1–4), indicating that the clonal repertoire is, in part, shared between both tissues. The degree of overlap was less pronounced between different individuals and did not appear to correlate with the tissue of origin. We thus deduce that at least part of the SARS-CoV-2–specific TCR repertoire is not tissue-specific (Figure 7A). The 10 most abundant clonotypes within each sample covered a percentage of cells that was correlated with the total number of cells, with all samples roughly showing the same degree of dependency, indicating that the heterogeneity of clonotypes is approximately similar in all samples regardless of tissue (Spearman’s ρ of cell number and percent covered by top clonotypes 0.91, *P* = 1.03 × 10^–5^; Figure 7B).

We next asked whether the clonotype had an impact on the transcriptomic identity of a cell. To this end, we identified all clonotypes with at least 4 cells, leading to a list of 9 different clonotypes. Highlighting the position of cells with a given clonotype in the UMAP projection revealed a strikingly close clustering in most cases, indicating that the gene expression profiles of these cells were much more similar than would be expected by pure chance (Figure 7C).

Next, in order to test which metadata are most influential in driving gene expression, we tested the transcriptomic correlation between all cells, and modeled the resulting Spearman’s correlation coefficients by the following parameters: same or different clonotype, cluster, tissue, and donor. Using Tukey’s honestly significant difference test, we determined the impact of same versus different metadata for each individual parameter, as well as any interaction of parameters, and found that sharing the same clonotype (or the same CDR3 sequence with a change in Spearman’s ρ of 0.074) had the largest positive effect on correlation of gene expression, followed by the cluster a cell was assigned to. In contrast, the tissue of origin had a smaller effect, and only a very small proportion of the correlation was driven by cells coming from the same donor (Figure 7D). Thus, the clonotype, i.e., the TCR sequence, was the best predictor of cells sharing similar transcriptomes.

Finally, we tested whether the overlap of clonotypes or CDR3 sequences between liver and paired blood could be caused by contaminating blood leukocytes in liver tissue. To that end, we calculated the odds ratios for a given clonotype or CDR3 sequence to be shared with blood for CD49^+^ or CD103^+^ tissue-resident memory T cell (Trm) versus CD49a^–^ or CD103^–^ nonresident (hypothetically contaminating) liver subsets. Notably, we did not observe significant changes in the clonotype or CDR3 overlap between the liver-derived tissue-resident versus nonresident populations with blood, respectively, as assessed by Fisher’s exact test (Figure 7E), indicating that a contamination was unlikely. We did not include transcripts for CD69 as a typical Trm marker in the previous analyses given its similar induction by peptide stimulation in both blood- and tissue-derived T cells.

## Discussion

So far, it had remained largely obscure whether and how cellular memory induced by intramuscular vaccination acquires residence in human tissues and how it may adapt to distinct local environments, particularly considering novel mRNA-based vaccines. Although the SARS-CoV-2 pandemic has stimulated research on numerous aspects of antiviral protection, the availability of human tissue specimens still constitutes the major limitation on comprehensive assessment of organ-specific immunity. Our study therefore provides pioneering data on tissue distribution, molecular signatures, and functional capacity of SARS-CoV-2–specific T cells generated after mRNA vaccination. Most importantly, particularly considering that samples were largely derived from virus-naive individuals with preexisting conditions, spike-specific CD4^+^ T cells were detectable approximately 3–4 months after vaccination in all nonlymphoid and lymphoid organ types analyzed except in tonsil. Besides peripheral blood, the highest responder rates were determined for bone marrow, in accordance with its role as survival niche of T cells specific for systemic pathogens (12). Since the aforementioned study and a related study (31) could not differentiate between vaccination- and infection-induced responses because of the live measles, mumps, and rubella (MMR) vaccine employed, there was no compelling indication so far that results could be extrapolated to mRNA vaccines.

Consistent with our recent findings on human kidney-derived bulk T cells (11), human renal peritumor tissue proved to be lymphopenic as compared with tumor specimens, supporting favorable quantification of vaccine-specific T cells in the latter. Whereas our previous report (11) showed similar frequencies of cytotoxic CD8^+^ T cells specific for persistent (Epstein-Barr virus [EBV], cytomegalovirus [CMV]) or seasonal (influenza) viruses in kidney and blood, we found enrichment of SARS-CoV-2 vaccination–induced CD4^+^ Th cells as a characteristic feature within nonlymphoid, as compared with lymphoid, organs. Importantly, frequencies of total vaccine-specific Th cells significantly declined with progressing vaccinee age in blood but not in paired nonlymphoid tissue samples. This observation also applied to specific CD45RO^+^CD62L^–^ Th cells, in agreement with the concept that maintenance of organ memory shows increased robustness (32). Stable age-independent persistence of immunological memory has been demonstrated for CMV- and influenza-specific CD8^+^ tissue-resident memory T (Trm) cells isolated, e.g., from human lung tissue (19). It needs to be considered, however, that T cells specific for persistent or recurring viruses are established early in life (33) and are subject to frequent reactivation in vivo, whereas longer-term maintenance of mRNA vaccine–induced tissue memory might critically depend on periodic boosters.

With respect to their specific organ adaptation, a substantial proportion of SARS-CoV-2–specific Th cells showed protein expression of the tissue residency–associated molecules CD69 and CD49a, whereas CD103^+^ T cells were less frequent and were mainly confined to the lung. This pattern broadly mirrors expression characteristics defined for bulk CD4^+^ T cells isolated from multiple human tissues, including lung and kidney (11, 34, 35), as well as of CD4^+^ T cells induced after SARS-CoV-2 infection (9). Based on our data, a segregation between lymphoid and nonlymphoid tissues was particularly evident for CD49a expression, showing an upregulation over blood only in liver, kidney, and lung. Interestingly, data from nasal tissue procured after mRNA vaccination support our observations in regard to specific T cell detection at distal sites in general and low frequencies of CD103^+^ cells in particular (36). Adequate comparisons between our findings and those of other studies on CD69 expression are limited by the fact that CD69 is uniformly upregulated by antigenic stimulation, a feature that we circumvent by stably staining cells before activation. Therefore, previous estimation of antigen-specific CD69^+^ Th cells from tissues has likely been biased (9, 37).

In principle, the concept of tissue memory entails optimized positioning of cells at potential future infection sites in concert with functional specializations (38). Among those, enhanced polyfunctionality has been determined as a distinct feature of tissue-derived lymphocytes as compared with their circulating counterparts (31, 35, 39), e.g., correlating with superior viral clearance after experimental influenza vaccination (40, 41). In that context, our cytokine expression data extend the notion that tissue-derived Th cells show functional modifications in comparison with their blood-derived counterparts: in accordance with cytokine expression in bulk T cells derived from various human organs (42), we found a significant enrichment of vaccine-induced polyfunctional Th cells in both nonlymphoid and lymphoid tissues.

To the best of our knowledge, cytokine production in either human CD49a^+^ or CD69^+^ tissue-derived CD4^+^ subsets has only been determined for bulk populations after polyclonal activation, such as in T cells isolated from human spleen and lung (34) or in our recent report on human kidney-derived Trm cells (11). We did not identify studies in which functions of both populations have been directly compared, particularly not in multiple solid organs. Consistent with our aforementioned report (11), we demonstrate that spike-specific CD49a^+^ cells show a trend toward higher frequencies of IL-2 producers as compared with CD49a^–^ T cells. In general, CD49a^+^ versus CD49a^–^ Trm cells appear to exhibit distinct functional adaptations to their anatomical niche as has been demonstrated for human skin (43). Our findings on differential IL-2 production in specific CD69^+^ versus CD69^–^ Th cells are opposed to bulk data from spleen or lungs (34). We can only speculate that bulk populations, consisting of memory cells specific for a multitude of infection- and vaccination-related antigens, show a functional pattern distinct from that of cells that have recently been differentiated in response to a single vaccination antigen. We have recently observed such functional alterations in recall (CMV, EBV, and flu) versus spike-specific responses in a SARS-CoV-2 vaccination study (4). Notably, such T cells reactive to 3 pooled recall antigens, thereby more resembling bulk responses, contained much higher frequencies of IL-2, IFN-γ, and TNF-α producers than spike-specific CD4^+^ T cells in virus-naive vaccines, suggesting distinct functional maturation kinetics that might also apply to different Trm subsets. We can only speculate, based on reports on murine CD8^+^ and CD4^+^ T cells in experimental infection (44, 45), that augmented IL-2 production in tissue-derived T cells might contribute to more robust memory formation.

So far, scRNA-Seq data of human vaccine-induced, tissue-derived CD4^+^ T cells are not available. Contrary to our expectations, we did not identify pronounced organ-specific transcriptome signatures of SARS-CoV-2 vaccine–induced CD4^+^ T cells. Instead, we observed a robust separation into 3 functionally distinct clusters that was largely independent of tissue origin. These data were further supported by TCR analysis, revealing that identical clonotypes are largely confined to the same cluster, indicating related functional programs associated with a given clone, even across (paired) tissue samples. Our finding that sharing the same clonotype had the largest positive effect on correlation of gene expression could be interpreted as indication that the vaccine-induced transcriptomic landscape, and thus the cell state and cluster annotation, are determined by the parent cell from which the TCR sequence was inherited — and not necessarily by tissue origin. Mechanisms of Trm cell ontogeny still remain incompletely addressed; however, the systemic residence memory differentiation model hypothesizes that T cells are transcriptionally marked based on their variable or identical TCR and therefore skewed toward either a Trm or circulating memory T cell (46–48). Our results mainly support such a concept, indicating that the TCR sequence is a strong determinant of the transcriptomic state in tissue-derived, vaccine-specific CD4^+^ T cells. More generally, it needs to be considered that the forces driving memory T cell diversification are likely different in infection versus vaccination settings, do not equally apply to CD8^+^ and CD4^+^ responses (9), and also depend on the inoculation site. In that context, data from a murine mRNA-based influenza vaccination model suggest that differentiation into CD8^+^ Trm cells could be induced by intramuscular priming, but is strongly boosted by secondary intranasal challenge (49). In the absence of such local booster immunization, as in our study, CD4^+^ Trm cells might not fully mature, providing an alternative hypothesis for why tissue origin did not dominantly drive cell clustering in our transcriptome analyses. The idea that tissue-resident T cells constitute an “inert” population that does not recirculate has recently been challenged by the demonstration of recruitment of human bone marrow Trm cells into the blood upon MMR revaccination (31). Accordingly, human skin Trm cells shuttling between tissue and blood are characterized by similar transcriptional programs (50), in line with our findings. These studies reflect the emergence of a more dynamic concept of tissue memory and associated molecular patterns that is currently discussed (38, 40); yet it has to be more substantiated with respect to vaccination-specific responses.

Our study has several limitations. Given that it relied on human surgery specimens, procurement of bona fide “healthy” human tissue was not feasible. Hence, we cannot completely rule out distinct effects of primary diseases of our organ donors on quantity and quality of tissue-derived lymphocytes. However, our comparative analysis of T cell responses in tumor versus nontumor patients, in line with comprehensive data on humoral and cellular immunity in solid cancer patients (51), suggests that the potential impact of preexisting conditions or treatment on vaccine-induced responses is likely small. Although we cannot estimate the exact impact of the stimulation approach on scRNA-Seq results, activation-induced marker–based (AIM-based) approaches for sorting of human antigen-specific T cells as a prerequisite for RNA-Seq analysis have been successfully employed in multiple settings (52, 53), including studies focusing on human Trm cell signatures (19). Activation-dependent transcriptional changes could theoretically be minimized by use of multimer-based cell purification, albeit restricting ensuing analyses by the concomitant selection of immunodominant peptides. Although we could identify vaccine-specific CD8^+^ T and B cells among multiple tissues, we were not able to similarly assess a possible dichotomy between nonlymphoid and lymphoid organs owing to limited responder rates or sample availability, respectively. Possible confounders with respect to differential systemic effects of primary disease or previous medication are excluded by our strategy to pairwise analyze blood and tissue specimens, assuming that both compartments are similarly affected by systemic preconditions. The same applies to the time passed since last vaccination. An impact of such bias could be further excluded by the fact that frequencies and functions of specific Th cells in blood and tissue remained stable over time since last vaccination, in accordance with other studies on long-term T cell maintenance after SARS-CoV-2 vaccination (54–56). A larger cohort would better compensate for the interindividual variation, and increasing of cell numbers for transcriptome analysis would allow a better representation of the cellular repertoire to a comparably large antigen as the SARS-CoV-2 spike protein.

In summary, we reveal here key features of novel mRNA vaccination–induced CD4^+^ Th lymphocytes with respect to their distribution across the human body, memory differentiation, age association, and functional adaptation that might be of relevance for the development of efficient vaccination strategies in the future.

## Methods

### Patients.

Macroscopic portions of tumor and/or most distant peritumor tissue as well as paired peripheral blood and serum samples were collected between October 2021 and October 2022 from patients diagnosed with a renal, liver, or lung tumor. Only a few patients with distant metastases and/or on chemotherapy (for less than 8 weeks before or after vaccinations or analysis) were included. Patients had no additional inflammatory diseases and did not receive immunosuppressive medication. Bone marrow was collected during spine surgery, and tonsils and spleens were collected following tonsillectomy and splenectomy. All patients were vaccinated against SARS-CoV-2 according to the national vaccination program and completed the 2- or 3-dose vaccination protocol with BNT162b2 (Comirnaty, BioNTech/Pfizer, 30 μg/dose) or with mRNA-1273 (Spikevax, Moderna/National Institute of Allergy and Infectious Diseases, 100 μg/dose). The interval between the shortest and the longest time point after last vaccination ranged from 19 to 265 days. Patient demographics, including time since last vaccination, are summarized in Table 1.

### Sample processing.

Serum samples were stored at –80°C. We typically received approximately 0.5 g (tonsil, kidney tumor) to 3 g (spleen, liver, lung, kidney peritumor) of tissue from surgery specimens that were immediately processed. To obtain single-cell suspensions, tissue was dissected into small pieces. Digestion medium was added, consisting of RPMI 1640 medium (Corning, Falcon, Kaiserslautern, Germany) supplemented with 0.3 mg/mL glutamine, 10% FCS (Gibco, Thermo Fisher Scientific, Darmstadt, Germany), 1% penicillin/streptomycin (Sigma-Aldrich, Merck, Darmstadt, Germany), 1 mg/mL collagenase II (Gibco), and 10 U/mL DNase I (Sigma-Aldrich, Merck). Samples were incubated for 45 minutes at 37°C while shaking. Reaction was stopped with medium without enzymes, and cells were passed through a 100 μm cell strainer (Corning). Thereafter, mononuclear cells (MNCs) were isolated with Leuko-Human Separating Solution (Genaxxon) by density gradient centrifugation and immediately cryopreserved; these 2 steps were also applied to peripheral blood mononuclear cells (PBMCs). Total mononuclear cell counts were in the range of 8 × 10^6^ (kidney tumor), 2 × 10^7^ (tonsil, lung, liver), and 10^8^ (spleen). Out of typically 9 mL bone marrow and peripheral blood, 1 × 10^7^ to 2 × 10^7^ MNCs were isolated.

### Assessment of humoral immunity.

Previous or current SARS-CoV-2 infection was assessed based on medical history and SARS-CoV-2 nucleoprotein-specific ELISA (Euroimmun). SARS-CoV-2 S1 domain–specific IgG was determined by ELISA (QuantiVac, Euroimmun). Serum samples with OD ratios of at least 1.1 (nucleoprotein-specific IgG) or at least 35.2 binding antibody units/mL (spike-specific IgG) were considered positive according to the manufacturer’s guidelines. OD ratios were calculated based on the ratio of the OD of the respective sample over the OD of the calibrator provided with the ELISA kit.

### Assessment of SARS-CoV-2 vaccine–specific B and T cells.

MNCs were thawed and washed twice in prewarmed RPMI 1640 medium containing 0.3 mg/mL glutamine, 100 U/mL penicillin, 0.1 mg/mL streptomycin, 20% FCS, and 25 U/mL benzonase (Santa Cruz Biotechnology Inc.). For identification of vaccine-specific T cells, 3 × 10^6^ to 5 × 10^6^ PBMCs per condition were stained with CD69-BV785 (FN50, BioLegend) for 20 minutes at room temperature to identify ex vivo expressing cells. CD69 staining was stable over the following stimulation period as demonstrated previously (11) and depicted in Supplemental Figure 1B. Thereafter, cells were rested for 2 hours at 37°C and stimulated or not for 16 hours with overlapping peptide 15-mers covering the complete SARS-CoV-2 spike protein (Alpha variant) at a final concentration of 0.5 μg/mL per peptide (JPT Peptide Technologies). In some experiments, PBMCs were additionally stimulated with a peptide pool encompassing the complete SARS-CoV-2 membrane and nucleocapsid proteins (Miltenyi Biotec). Brefeldin A (Sigma-Aldrich, St. Louis, Missouri, USA) was added after 2 hours. T cells were identified as CD3^+^CD19^–^CD14^–^ live (“dump^–^”) single lymphocytes. Vaccine-specific CD4^+^ Th cells were identified based on CD137 and CD40L, whereas specific CD8^+^ T cells were detected based on CD137 and IFN-γ coexpression with the gating strategy depicted in Supplemental Figure 1A.

A response was defined as positive when stimulated cultures contained at least 2-fold higher frequencies of CD137^+^CD154^+^ CD4^+^ T cells or CD137^+^IFN-γ^+^ CD8^+^ T cells as compared with the respective unstimulated control with at least 20 events, as reported previously (4, 16). For assessment of polyfunctionality, samples with at least 40 CD137^+^CD40L^+^ cells were included. For surface labeling, antibodies listed in Supplemental Table 1 were used. After surface staining, cells were fixed with FACS Lysing Solution (BD Biosciences) followed by permeabilization in FACS Perm II Solution (BD Biosciences) and stained intracellularly with antibodies as summarized in Supplemental Table 1.

B cells were detected within 5 × 10^6^ to 10 × 10^6^ MNCs by flow cytometry and gated as CD19^+^CD3^–^CD14^–^CD56^–^ live (“dump^–^”) single lymphocytes. SARS-CoV-2–specific B cells were identified as shown previously (57) by double staining with recombinant receptor-binding domain (RBD) protein (Alpha variant, R&D Systems) coupled to Alexa Fluor 488 and recombinant full spike protein coupled to biotin (Alpha variant, R&D Systems), with the latter detected by streptavidin-APC (BioLegend). The gating strategy is depicted in Supplemental Figure 10. For further flow cytometric surface marker expression analysis, antibodies depicted in Supplemental Table 2 were used. Data were acquired using a BD FACS Fortessa X20 (BD Biosciences) with DIVA software v8.0.7.

### FACS data analysis and statistics.

FACS data analysis was conducted with FlowJo 10 (BD Biosciences). Frequencies of spike-specific T cells were background subtracted (background = unstimulated control). Coexpression of cytokines was quantified by Boolean gating in FlowJo. Statistical analysis and graph preparation were performed in GraphPad Prism 8. Data distribution was assessed using the Kolmogorov-Smirnov test. Depending on whether distribution was normal, 1-way ANOVA (with Holm-Šidák post hoc), Kruskal-Wallis test (with Dunn’s post hoc), or Friedman test was chosen for multiple comparisons. For 2-group comparisons, unpaired *t* test (2-tailed) or Mann-Whitney test (2-tailed) was used. The relationship between 2 variables was examined by simple linear regression analysis. For analysis of contingency tables, Fisher’s exact test was applied. In all tests, a *P* value less than 0.05 was considered significant.

### Enrichment of vaccine-specific CD4^+^ T cells and scRNA-Seq.

For single-cell transcriptome (scRNA-Seq) analysis, 10^7^ MNCs from peripheral blood, liver, lung, and bone marrow were stimulated for 16 hours with SARS-CoV-2 spike peptide mix in the presence of anti-CD40 (1 μg/mL; HB14, Miltenyi Biotec) to retain CD154 on the surface of specific CD4^+^ T cells (58). Thereafter, antigen-reactive cells were surface-stained with anti-CD154–PE (24-31, BioLegend) and magnetically enriched using anti-PE nanobeads (BioLegend) over MACS LS columns (Miltenyi Biotec). Spike-specific CD3^+^CD4^+^dump^–^CD154^+^CD137^+^ cells were further FACS-purified in single-cell mode to typically >97% purity (exemplarily depicted in Supplemental Figure 1A) into PBS/BSA buffer containing round PCR tube lids on an Aria Fusion cell sorter (BD Biosciences). To minimize cell loss related to small numbers of specific T cells, samples were individually spiked with dump^–^CD3^–^CD4^–^CD8^–^CD56^+^ natural killer (NK) cells from the same sample, resulting in a total of 5,000 cells per sort. The cell suspension was loaded into a 10X Chromium Controller using 10X Genomics Chromium Next GEM Single Cell V(D)J Reagent Kit v1.1, and the subsequent reverse transcription, cDNA amplification, and cDNA library preparation were performed according to the manufacturer’s instructions. 5′ gene expression libraries and target-enriched libraries (TCR, for human T cells) were quantified by Qubit 4.0 Fluorometer (Thermo Fisher Scientific), and quality was checked using 4200 Tapestation with High Sensitivity DNA kit (Agilent). Libraries were then pooled at a 10:1 ratio (5′ gene expression library/target-enriched library). Sequencing of the pooled library was performed in paired-end mode with S Prime flow cells (SP) (2 × 50 cycles kit) using a NovaSeq 6000 sequencer (Illumina).

### scRNA-Seq and statistics.

Primary analysis of TCR and 5′ gene expression libraries was performed using Cell Ranger V(D)J 6.1.2 (10X Genomics). Reference build 5.0.0 (VDJ) and GRCh38-2020A (gene expression) were used. Conserved clonotypes within cells from a single donor were identified using Cell Ranger aggr, while downstream analysis of gene expression data was performed on the unaggregated samples. For secondary analysis, Seurat 4.2.1 (59) was used. After pre-filtering, excluding any cell with fewer than 200 genes expressed or more than 10% mitochondrial reads, as well as any gene expressed in fewer than 3 cells, gene expression was normalized to 10,000 reads per cell. As sequenced cells were a pool of NK and CD4^+^ T cells, the latter were extracted using a filter defining any cell with at least 1 read of CD4 or a TCR sequence and no reads of *FCGR3A* as a CD4^+^ T cell. Samples were then integrated using Harmony 0.1.0 (60). After principal component analysis, nearest-neighbor graph calculation, and Leiden clustering (61) with a resolution of 0.1, four clusters of cells were identified. The smallest cluster showed transcriptional patterns akin to NK cells and was thus deemed a likely contamination. Any further analyses were performed using the 3 larger clusters (clusters 0, 1, and 2). Differentially expressed genes were identified using the FindMarkers and FindAllMarkers functions (Harmony 0.1.0) with Wilcoxon’s test. Pathway enrichment analyses were performed using MetaScape (62). For RNA velocity analyses, spliced and unspliced count matrices were generated using velocyto CLI (version 0.17.17). Velocity was then calculated and projected onto UMAP using velocyto.R (version 0.6). For analyses by tissue, the data set was split before velocity calculation. To estimate the skewing of cell state distribution of clonotypes, a Monte Carlo simulation was run for each clonotype to determine the tail probability of observing a distribution that was as skewed as or more skewed than the one observed. The *P* value across clonotypes was then calculated as their joint distribution.

### Study approval.

The study was approved by the local Ethics Committee of the Charité – Universitätsmedizin Berlin (EA4/066/19, EA1/353/16, EA4/115/21) and University Hospital Leipzig (322/17-ek, 237/22-ek) and was conducted in compliance with the Declarations of Helsinki and Istanbul. All patients provided written informed consent.

### Data and materials availability.

All cellular data needed to evaluate the conclusions in the paper are present in the paper or the supplemental material. In order to ensure participant confidentiality, raw data are available under controlled access in the European Genome-Phenome Archive repository (EGAS50000000045). This study did not use any unique codes, and all analyses were performed in R and Python using standard protocols from previously published packages as indicated. Values for all data points in the figures can be found in the Supporting Data Values file. Requests for materials should be directed to the corresponding authors.

## Author contributions

VP, AS, CC, and KK designed experiments. VP, AS, LT, LMLT, JS, AH, KJ, CL, CS, and SL performed experiments. VP, AS, SL, CC, and B Kittner analyzed the data. VP, AS, SL, CC, and KK interpreted the data. GD, DS, CG, CB, PJ, AM, SE, B Kirzinger, TM, KB, DL, JK, JB, B Kittner, FR, FF, MH, SW, NL, ES, and HS provided tissue and blood samples and clinical data, analyzed clinical data, and consulted and discussed the manuscript. HH and VMH provided tissue and blood samples and analyzed clinical data. AS, CC, and KK supervised the project. VP, AS, SL, and KK wrote the manuscript. All authors reviewed and approved the final manuscript. The order of the equally contributing first authors was determined based on consensus.

## Supplementary Material

Supplemental data

Supporting data values

## Figures and Tables

**Table 1:**
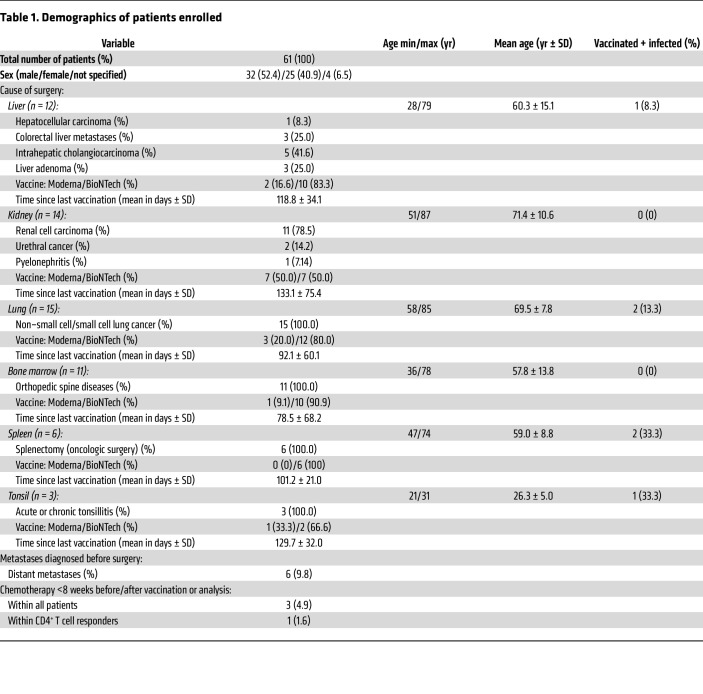
Demographics of patients enrolled

## Version 1 10/10/2023

In-Press Preview

## Version 2 12/15/2023

Electronic publication
