# Supplementary material for: SARS-CoV-2 mRNA vaccination–induced immunological memory in human nonlymphoid and lymphoid tissues
Source: J Clin Invest. 2023 Dec 15;133(24):e171797. doi: 10.1172/JCI171797 (PMC10721158; doi:10.1172/JCI171797)
Supplement: Supplemental data [file jci-133-171797-s064.pdf]

Proß, Sattler, Lukassen et al. Supplemental Figure 1

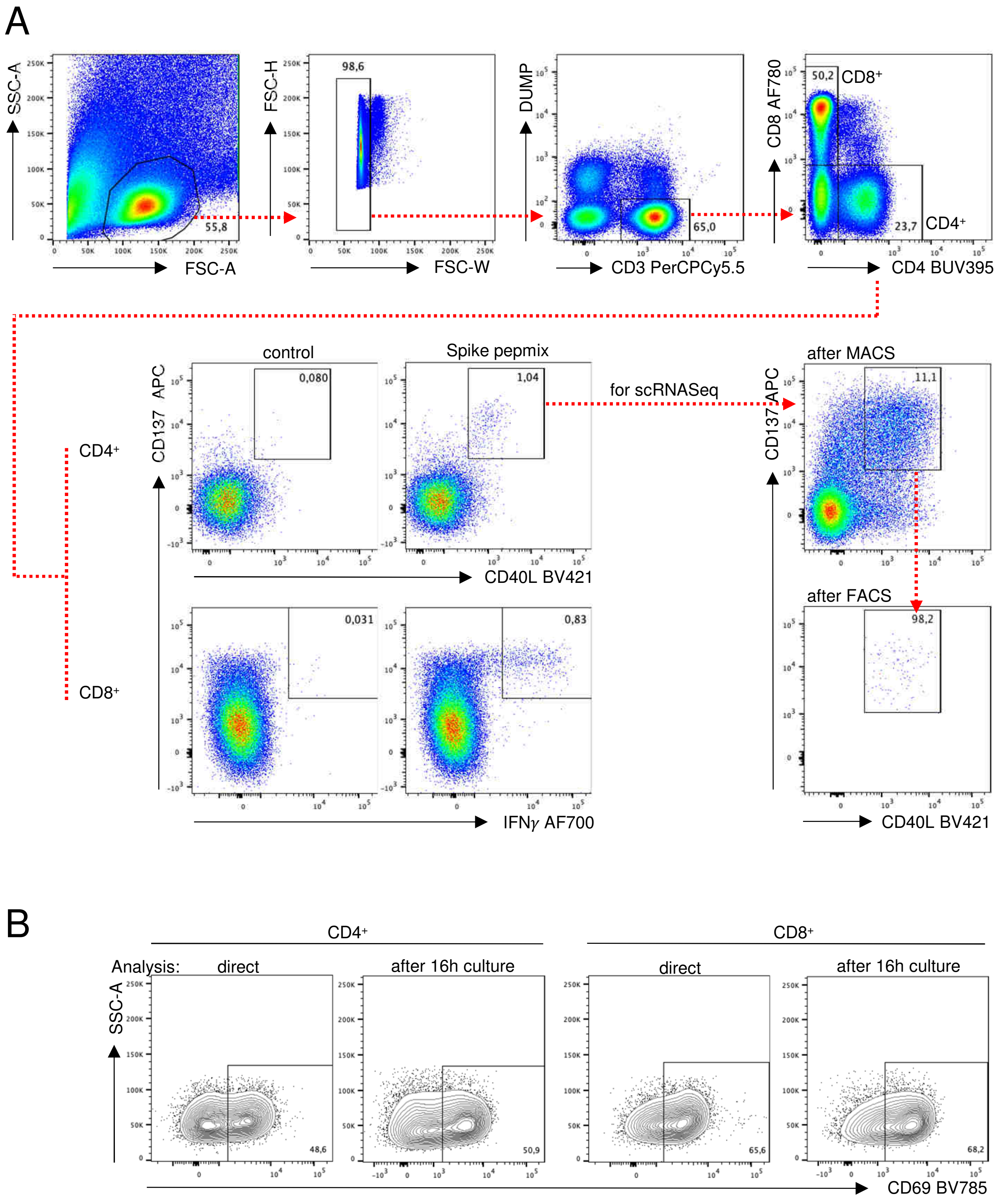

Supplemental Figure 1. Gating strategies for identification of SARS-CoV-2 vaccine-specific T cells. (A) Exemplary, liver derived MNCs were stimulated or not with Spike-peptide mix for 16 h as indicated. Live single CD14⁻CD19⁻CD3⁺ specific CD4⁺ Th cells were identified by FACS according to co-expression of CD137 and CD40L. Doublets were excluded based on FSC-W/FSC-H signals. For scRNASeq analysis, CD40L⁺ cells were magnetically enriched, followed by FACS sort of live CD4⁺CD137⁺CD40L⁺ cells to typically >97 % purity. Spike-specific CD8⁺ T cells were detected based on CD137 and IFNγ co-expression. (B) Exemplary, lung-derived MNCs stained with CD69 BV785 showed no relevant loss of CD69 signal when analyzed by FACS after 16 h of cultivation as compared to direct analysis.

Proß, Sattler, Lukassen et al. Supplemental Figure 2

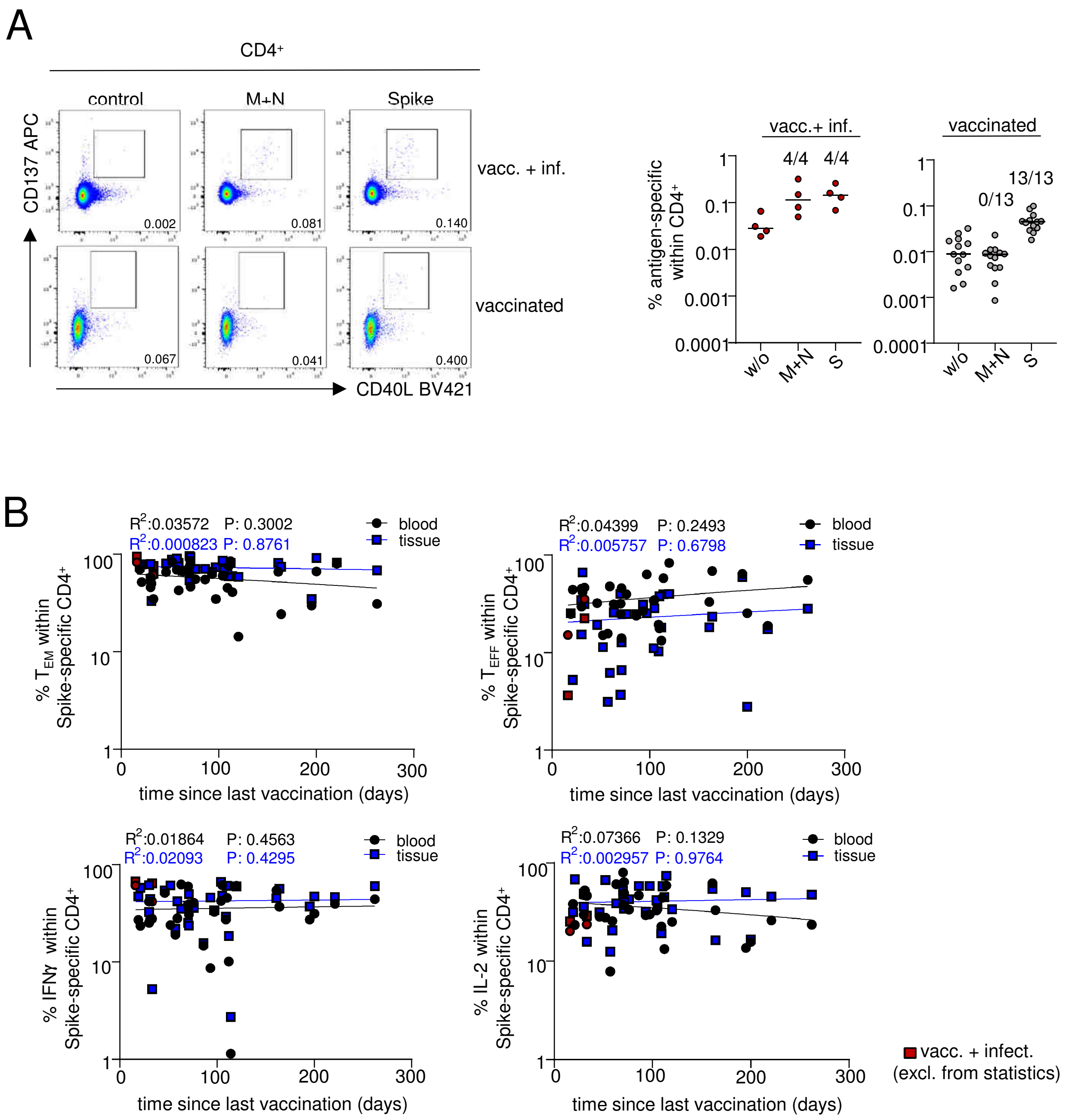

Supplemental Figure 2. T-cell based assessment of previous SARS-CoV2-infection and impact of time since last vaccination on Spike-specific responses. (A) Exemplary dot plots (left) and summary (right) of specific CD4+ T cell responses in PBMC after SARS-CoV2 membrane plus nucleocapsid (M+N) or Spike peptide mix stimulation in probands considered vaccinated only (n=13) or vaccinated and infected (n=4). Numbers above graphs (right) indicate individuals with a response to the respected stimuli. (B) Correlation analyses of blood and tissue derived frequencies of Spike-specific effector memory (upper left), effector (upper right), IFNγ+ (lower left) or IL-2+ (lower right) CD4+ T cells with time since last vaccination. BM: n=10, spleen: n=3, liver: n=8, kidney: n=8, lung: n=7; simple linear regression. Red symbols identify vaccinated individuals with a history of SARS-CoV2 infection that are excluded from statistics.

Proß, Sattler, Lukassen et al. Supplemental Figure 3

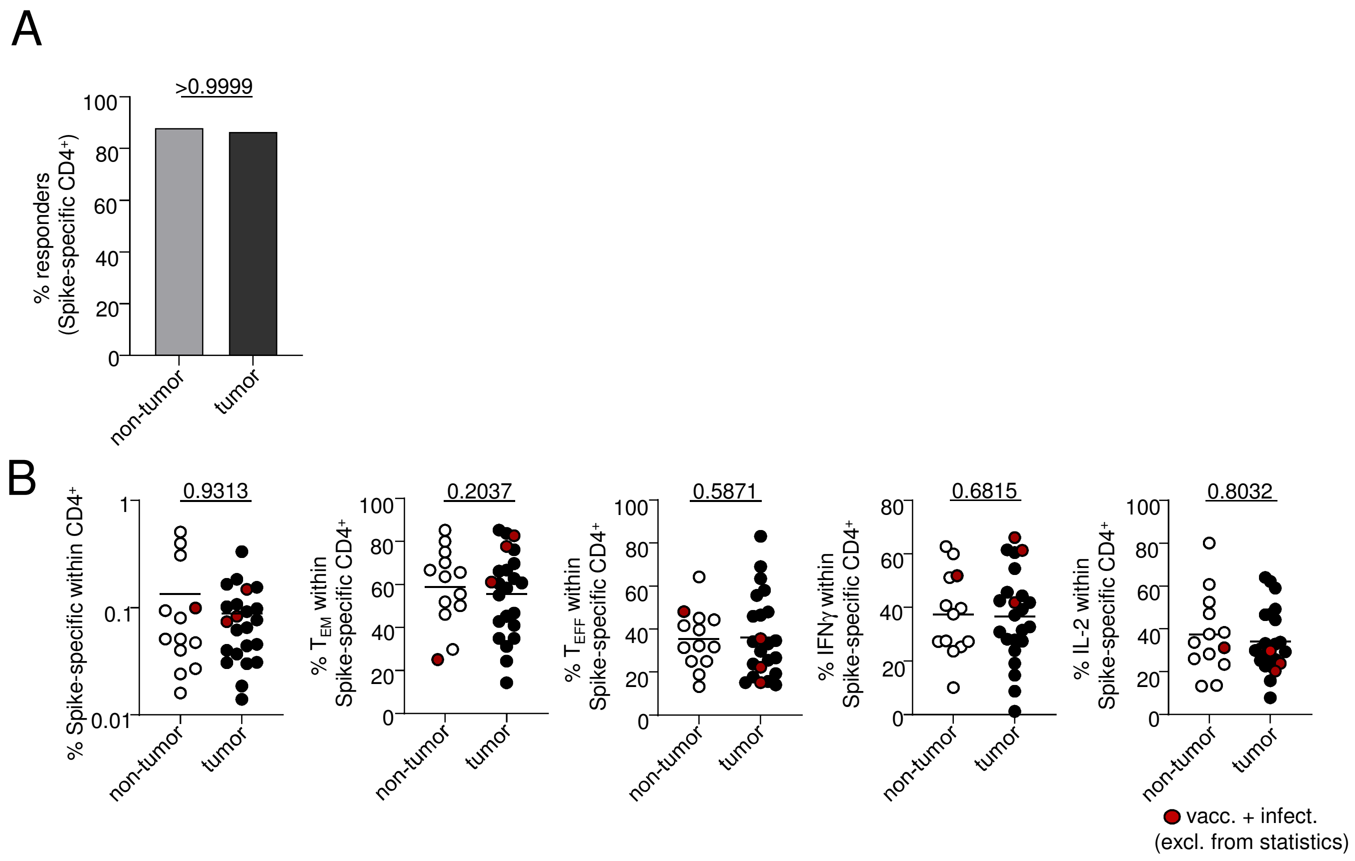

Supplemental Figure 3. Impact of patient precondition on quantity and/or quality of vaccine-specific CD4+ T cells. (A) Spike-specific CD4+ T cell responder rates based on blood of non-tumor (spleen, BM) vs. tumor (liver, lung, kidney) patients. Statistically significant differences were tested with the two-sided Fisher's exact test. (B) Frequencies of overall Spike-specific blood-derived CD4+ T cells, of those with an effector memory phenotype or expressing cytokines as depicted, in patients stratified as in (A). Non-tumor: n=13, tumor: n=23. Red symbols identify vaccinated individuals with a history of SARS-CoV2 infection. Only datasets where paired blood and tissue samples were available are included, allowing comparability with the donors included in the mostly pairwise comparisons throughout the manuscript.

Proß, Sattler, Lukassen et al. Supplemental Figure 4

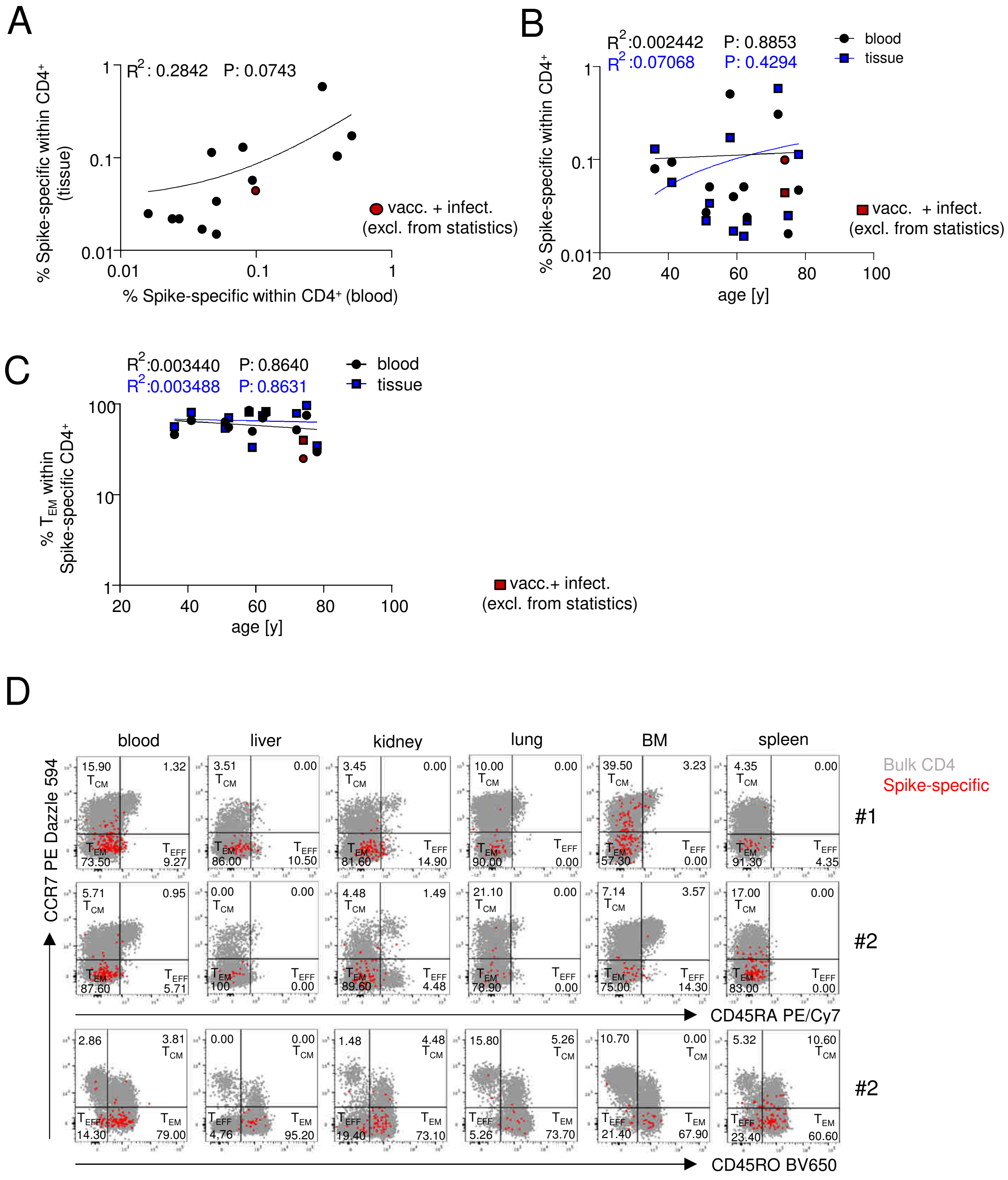

Supplemental Figure 4. Correlation analyses for Spike-specific, lymphoid organ-derived CD4+ Th cells and memory phenotype according to CCR7 expression. Correlation analyses were performed as in Figure 1H and I for frequencies of specific Th cells from lymphoid organs (BM, spleen) against those detected in blood (A) or of both against age (B). (C) Correlation analysis was performed as in Figure 2C for frequencies of antigen-specific T_{EM} from lymphoid organs. BM=10; spleen=3; simple linear regression. Red symbols identify vaccinated individuals with a history of SARS-CoV2 infection that are excluded from statistics. (D) Memory phenotype classification for two sets (#1 and #2) of the depicted specimen types based on CD45RA/CCR7 staining of Spike-specific Th cells. For comparison purposes, the combination of CD45RO/CCR7 is included for #2. Bulk CD4+ T helper cells in grey are overlaid by the Spike-specific population in red.

Proß, Sattler, Lukassen et al. Supplemental Figure 5

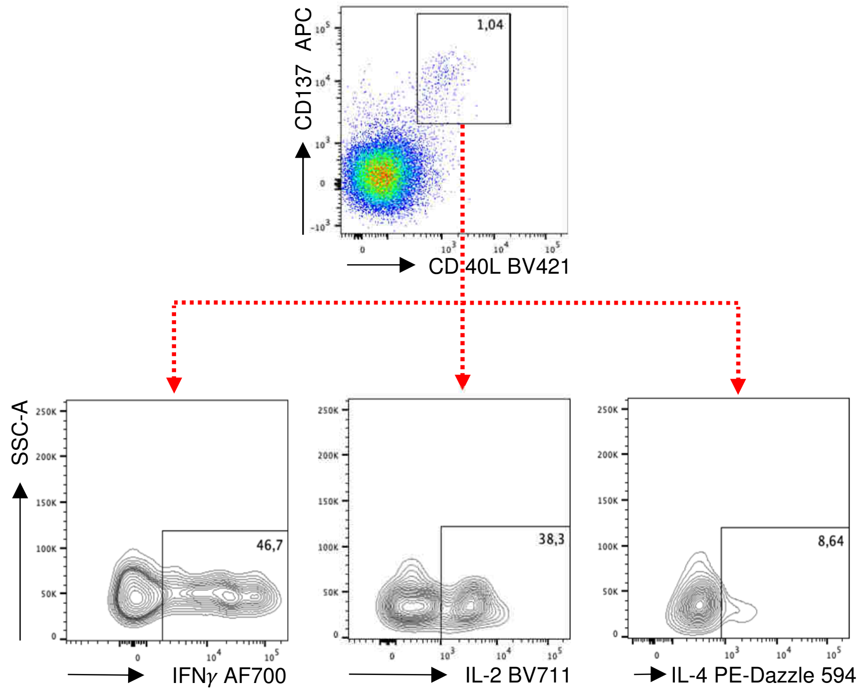

Supplemental Figure 5. Gating strategy for identification of cytokine-producing SARS-CoV2 vaccine-specific T cells. (A) Specific CD4$^+$CD137$^+$CD40L$^+$ T cells were further analyzed for expression of IFNγ, IL-2 and IL-4. Gates were applied according to the respective unstimulated controls.

Proß, Sattler, Lukassen et al. Supplemental Figure 6

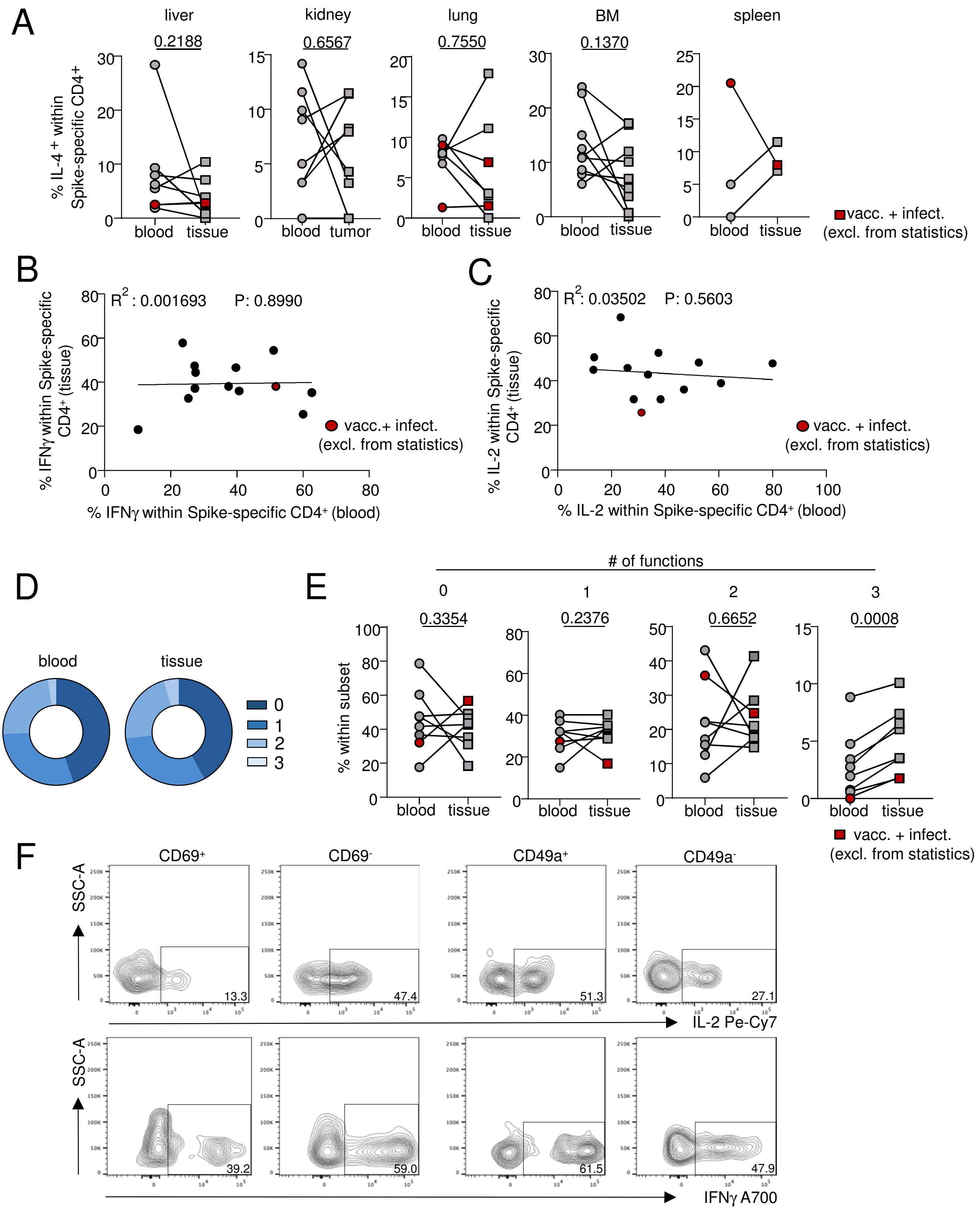

Supplemental Figure 6. Extended functional features of Spike-specific CD4+ Th cells. (A) FACS-based expression analysis of IL-4 in Spike-specific Th cells in paired tissue and blood samples. Liver: n=8, Wilcoxon test; kidney: n=8, paired t test; lung: n=7, paired t test; BM: n=10, Wilcoxon test; spleen: n=3, paired t test. (B) Correlation analyses were performed as in Figure 4B and C for frequencies of specific IFNγ (B) and IL-2 (C) positive Th cells from lymphoid organs (BM, spleen) against those detected in blood. BM: n=10; spleen: n=3; simple linear regression. (D and E) Polyfunctional features of specific lymphoid tissue (BM) derived Th cells as in Figures 4D and E. Statistically significant differences were tested with paired t test. (F) Exemplary plots from kidney tumor tissue illustrating differential cytokine production associated with CD69/CD49a expression, related to Figure 4F. Red symbols identify vaccinated individuals with a history of SARS-CoV2 infection that are excluded from statistics.

Proß, Sattler, Lukassen et al. Supplemental Figure 7

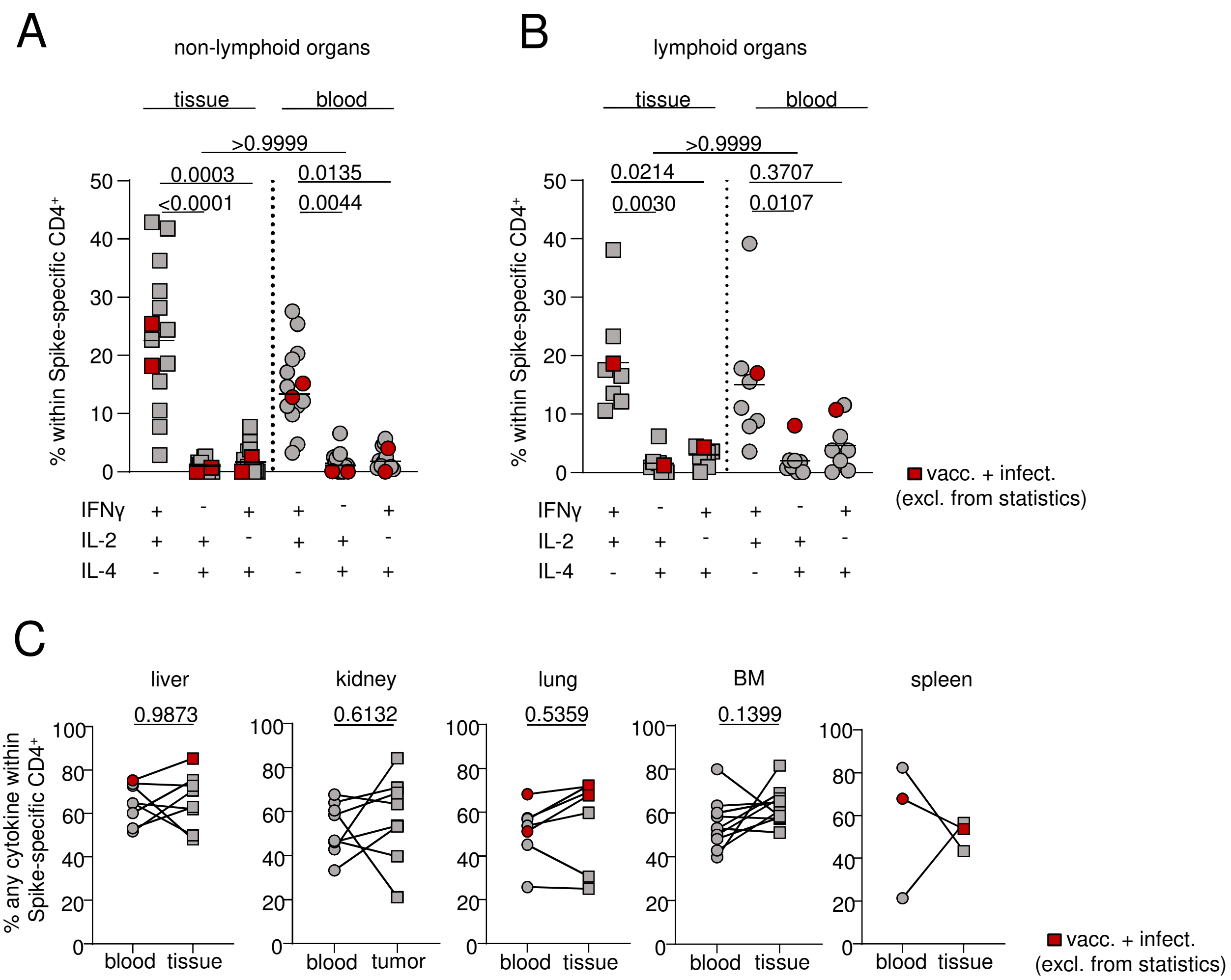

Supplemental Figure 7. Cytokine co-expression and global cytokine production in SARS-CoV2-specific CD4+ T cells. Co-expression analysis of IFNγ, IL-2 and IL-4 in Spike-specific CD4+ T cells from (A) non-lymphoid and (B) lymphoid organs vs. paired blood samples, respectively (both Friedman test). (C) Frequencies of spike-specific CD4+ T cells expressing any of the cytokines IFNγ, IL-2 and/or IL-4 in the depicted tissues vs. paired blood samples (all paired t test). Liver: n=8, kidney: n=8, lung: n=7, BM: n=10 and spleen: n=3 in (A)-(C).

Proß, Sattler, Lukassen et al. Supplemental Figure 8

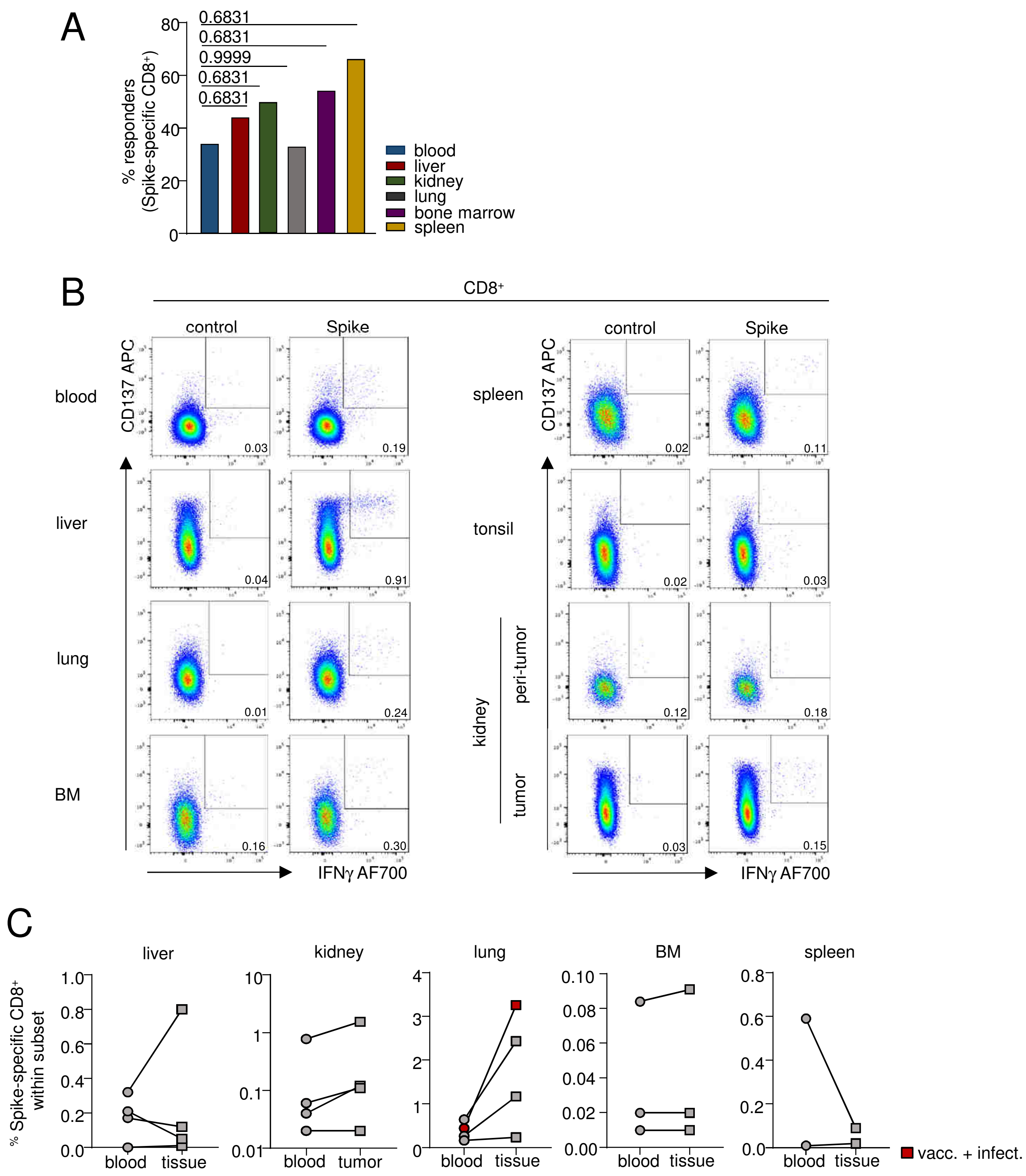

Supplemental Figure 8. Quantification of vaccine-specific CD8+ T cell responses. (A) Portions of individuals showing specific CD8+ T cells responses within the depicted organs. Statistically significant differences were tested with two-sided Fisher's exact test. (B) Exemplary plots and (C) summary showing vaccine-specific CD137+IFNγ+ CD8+ T cell quantities from the indicated organs as identified by FACS. Liver: n=4, kidney: n=4, lung: n=4, BM: n=3, spleen: n=2. Red symbols identify vaccinated individuals with a history of SARS-CoV2 infection.

Proß, Sattler, Lukassen et al. Supplemental Figure 9

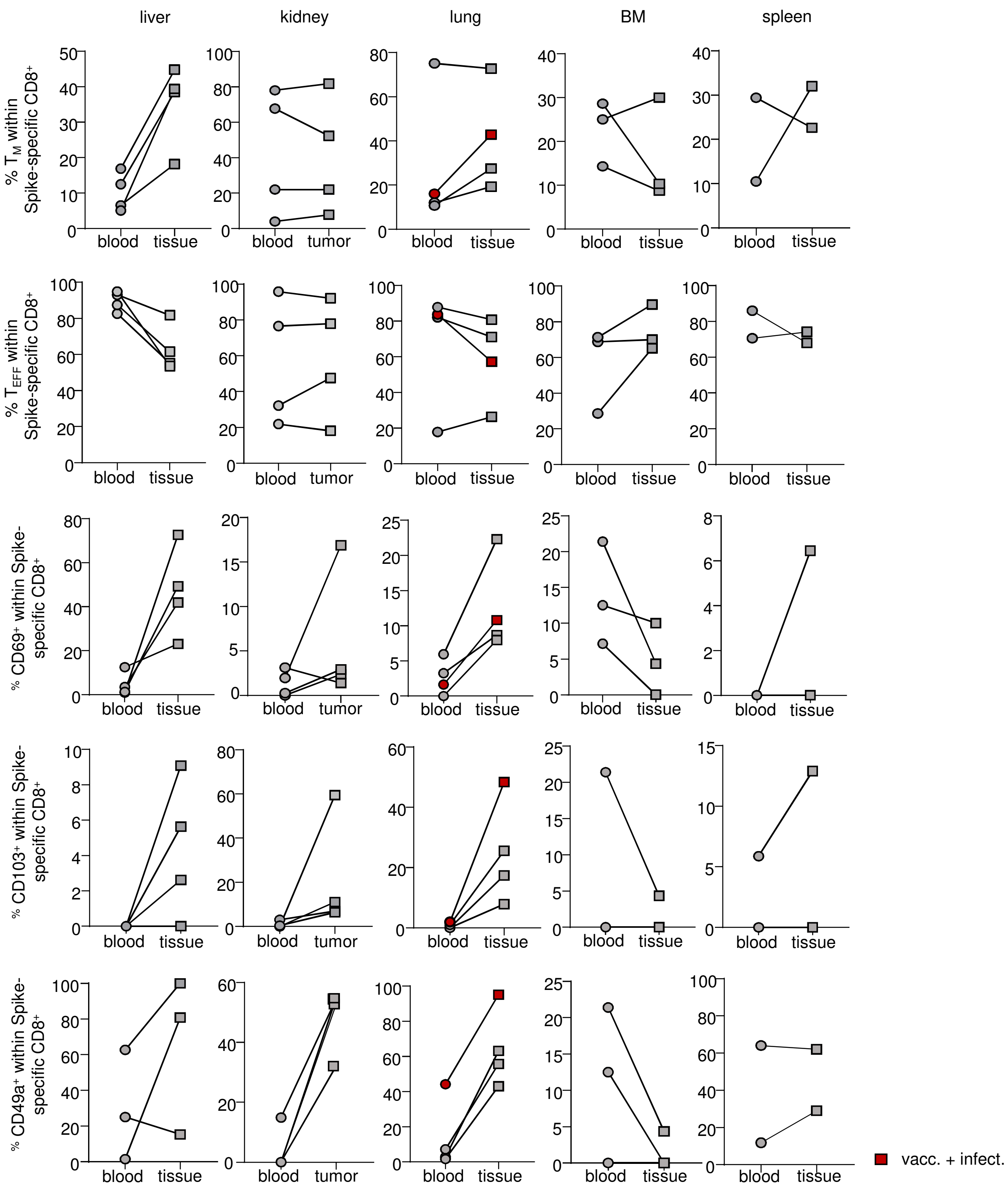

Supplemental Figure 9. Extended phenotyping of Spike-specific CD8⁺ T cells. Specific CD8⁺ T cells were identified as described before and further analysed for expression of memory (CD45RO, CD62L) or tissue residency/retention (CD69, CD103, CD49a) associated molecules in paired blood and organ samples as indicated. Liver: n=3/4, kidney: n=4, lung: n=4, BM: n=3, spleen: n=2. Red symbols identify vaccinated individuals with a history of SARS-CoV2 infection.

Proß, Sattler, Lukassen et al. Supplemental Figure 10

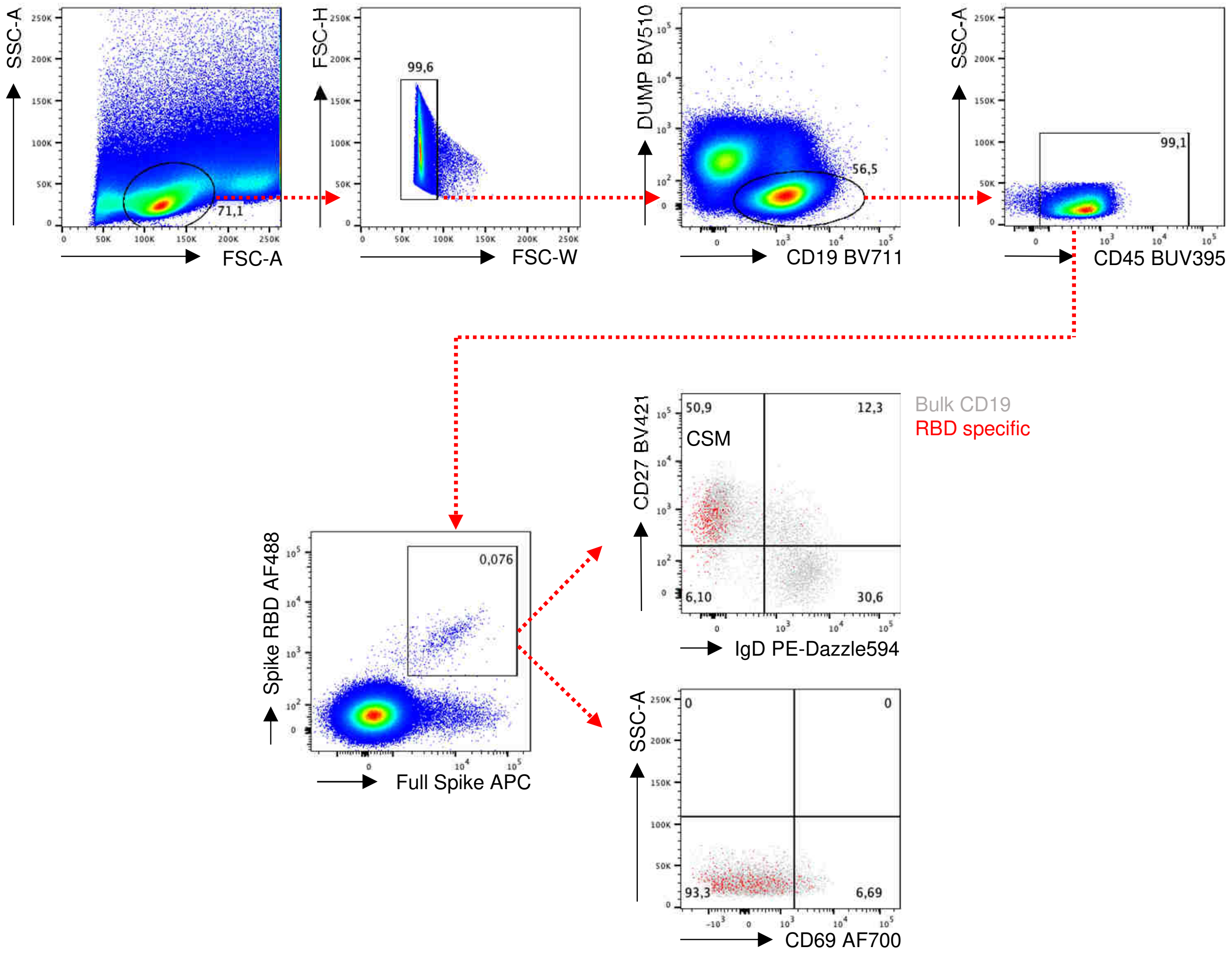

Supplemental Figure 10. Gating strategy for assessment of SARS-CoV2-specific B cells. Spike RBD-specific live single CD14$^-$CD56$^-$CD3$^-$ ("dump" negative) CD19$^+$ B cells were exemplarily identified in MNC from tonsil by flow cytometry based on co-staining with recombinant Spike RBD-FITC and recombinant full Spike-APC. Specific cells were further analyzed for memory differentiation (CD27, IgD), where isotype class-switched memory cells (CSM) were CD27$^+$IgD$^-$, or CD69 expression.

Proß, Sattler, Lukassen et al. Supplemental Figure 11

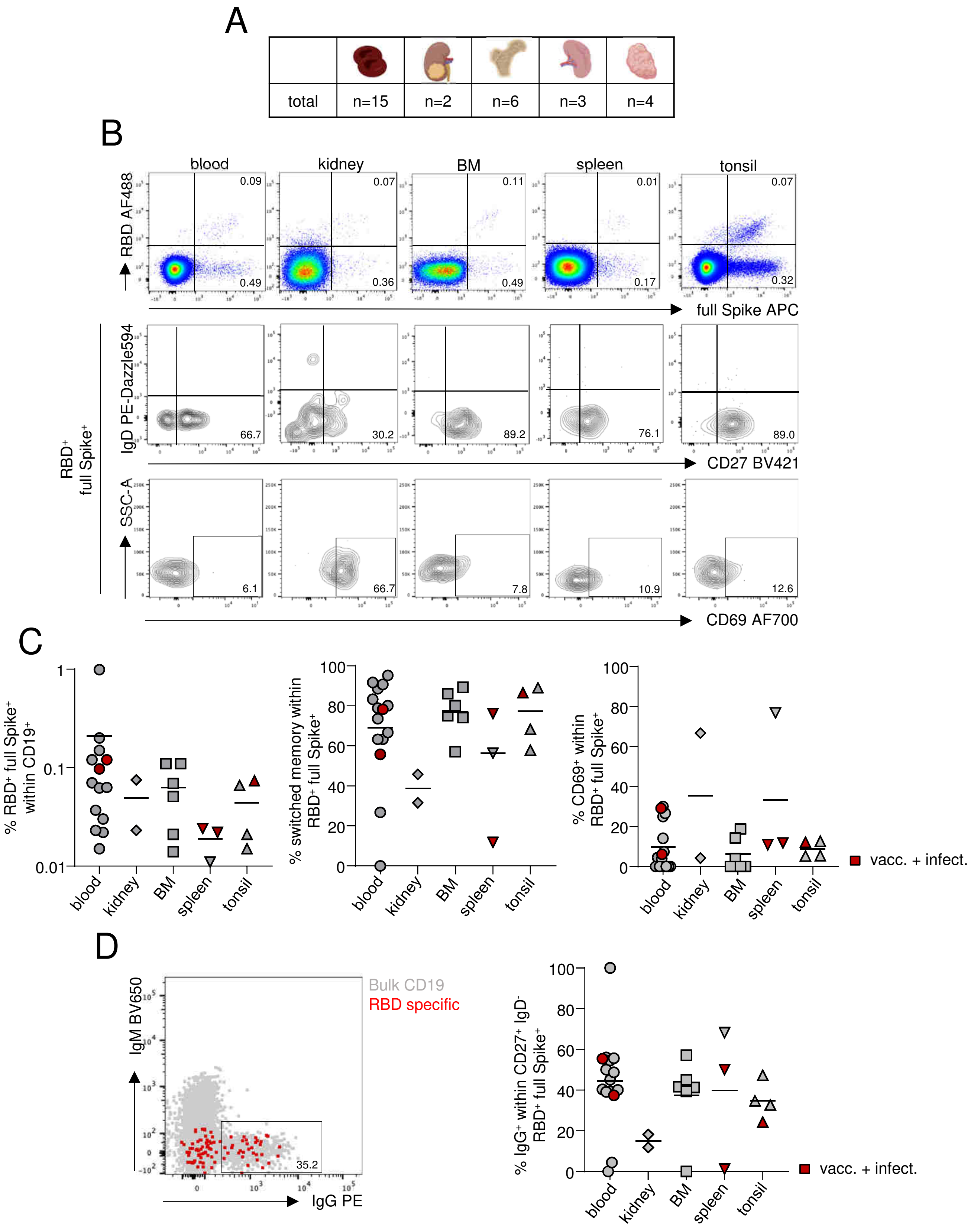

Figure 11. Quantification of SARS-CoV2-vaccine-specific B cells in tissues. (A) Summary of specimen types and their quantities included for specific B cell analysis. (B) Exemplary plots illustrating frequencies of Spike-RBD-specific B cells in the indicated organs (upper panel), their memory phenotype according to CD27 and IgD expression (middle panel) and CD69 expression (lower panel). (C) summarizes data for the indicated specimen types. (D) Exemplary dot plot (left) and summary (right) for frequencies of IgG⁺ B cells within the SARS-CoV2 RBD-specific $CD27^{+}IgD^{-}$ memory compartment in the depicted specimen types. Blood: n=15, kidney: n=2, bone marrow: n=6, spleen: n=3, tonsil: n=4 for all analyses. Red symbols identify vaccinated individuals with a history of SARS-CoV2 infection. Given the small sample size, statistical analyses were not conducted

Proß, Sattler, Lukassen et al. Supplemental Figure 12

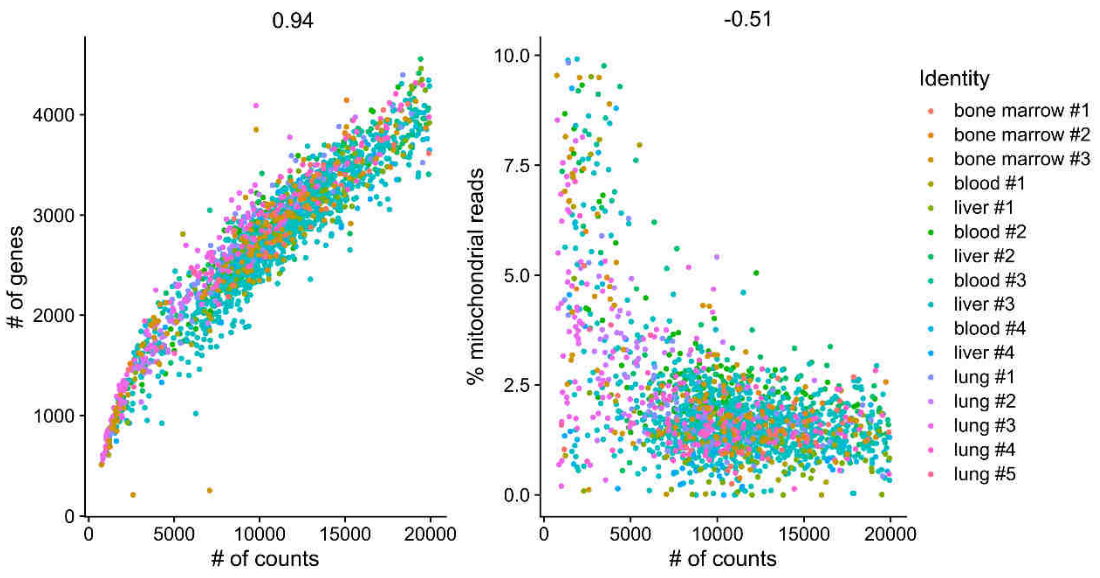

Supplemental Figure 12. Scatter plots of per-cell quality control metrics as depicted for single-cell RNA-Seq data. Samples are color-coded, Pearson correlation coefficients are indicated above the panels.

Proß, Sattler, Lukassen et al. Supplemental Figure 13

cluster 0 – „cytokine signaling“ (226 genes up-regulated)

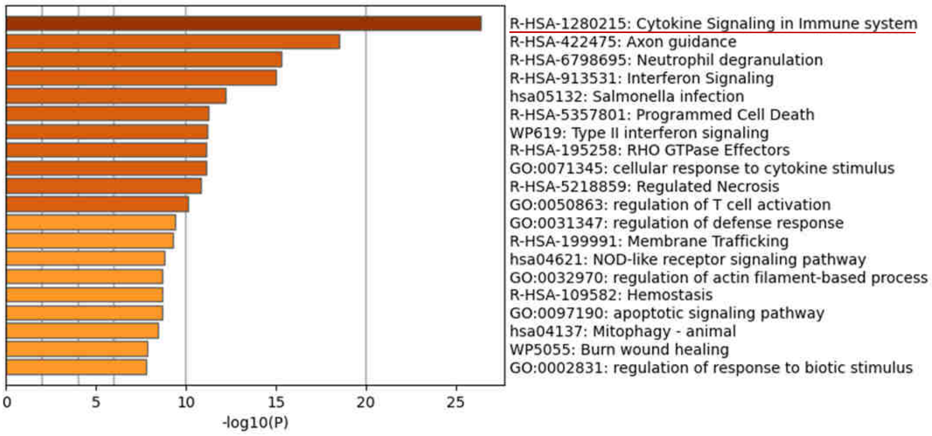

cluster 1 - „ribosomal biogenesis“ (437 genes up-regulated)

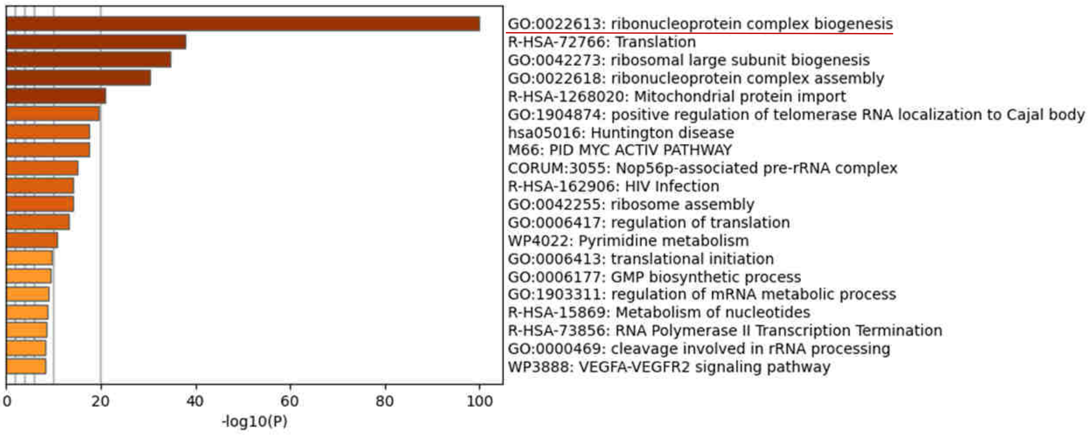

cluster 2 – „NEAT1“ (194 genes up-regulated)

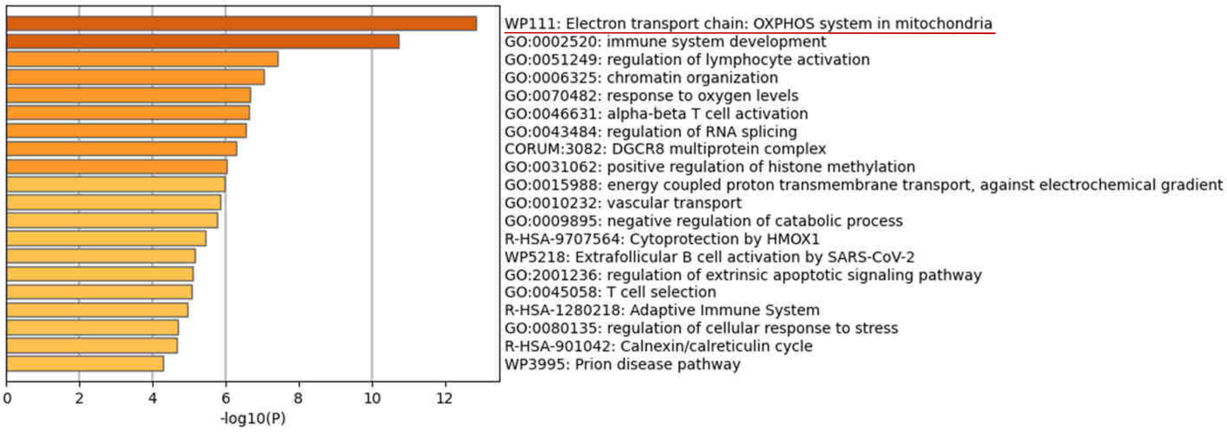

Supplemental Figure 13. **Pathway enrichment analysis.** Top enriched pathway analysis of transcripts enriched in cluster 0, 1 and 2. Significantly overrepresented biological processes (p≤0.05) in each cluster were plotted against Enrichment Score (calculated as -log10 p-value).

Supplemental Table 1. Antibodies used for T cell analysis

Molecule	Clone	Fluorochrome	Manufacturer	Catalog Number
Surface				
CCR7	GO43H7	PE-Dazzle 594	BioLegend	353235
CD3	SK7	PerCP/Cy5.5	Biolegend	344808
CD4	SK3	BUV395	BD	563550
CD8	SK1	APCeFluor780	Thermo Fisher	47-0087-42
CD14	M5E2	BV510	Biolegend	301842
CD19	H1B19	BV510	Biolegend	302242
L/D Zombie	-	Aqua (BV510)	Biolegend	423101
CD45RA	HI100	PE/Cy7	BioLegend	304125
CD45RO	UCHL1	BV650	Biolegend	304232
CD49a	TS2/7	PE/Cy7	Biolegend	328311
CD62L	DREG-56	BV605	BD	304834
CD69	FN50	BV785	Biolegend	310932
CD103	Ber-ACT8	PE	Biolegend	350206
Intracellular				
CD137	4B4-1	APC	Biolegend	309810
CD40L	24-31	BV421	Biolegend	310824
IFNγ	4S.B3	FITC	Biolegend	502506
IL-2	MQ1-17H12	BV711	Biolegend	500345
IL-4	MP4-25D2	PE-Dazzle594	Biolegend	500832

Supplemental Table 2. Antibodies used for B cell analysis

Molecule	Clone	Fluorochrome	Manufacturer	Catalog Number
CD3	UCHT1	BV510	Biolegend	344828
CD14	M5E2	BV510	Biolegend	301842
CD56	5.1H11	BV510	Biolegend	362534
L/D Zombie	-	Aqua(510)	Biolegend	423101
CD45	HI30	BUV395	BD	563792
CD19	SJ25C1	BV711	Biolegend	363022
CD27	M-T271	BV421	Biolegend	356417
IgD	IA6-2	PE-Dazzle594	Biolegend	348240
IgG	IS11-3B2.2.3	PE	Miltenyi Biotec	130-119-878
Spike RBD	-	AF488	R&D Systems	AFG10500-020
Full Spike	-	Biotin	R&D Systems	BT10549-050
Streptavidin	-	APC	Biolegend	405207
CD69	FN50	AF700	Biolegend	310922
